# Shared Versus Unique Features of Neural Activation During Cognitive Flexibility Across Restrictive Eating Disorder Presentations

**DOI:** 10.1002/eat.24599

**Published:** 2025-11-11

**Authors:** Adrienne L. Romer, Lauren Breithaupt, Meghan Slattery, Felicia Petterway, Lauren Lindman, Jason Scott, Meghan Lauze, Mia Cravitz, Zara Poon, Siddarth Seenivasa, Sarah Naticchia, Kristin N. Javaras, David Alperovitz, Jennifer J. Thomas, Elizabeth A. Lawson, Diego A. Pizzagalli, Franziska Plessow, Poornima Kumar, Madhusmita Misra, Kamryn T. Eddy

**Affiliations:** ^1^ Department of Psychology Virginia Polytechnic Institute and State University Blacksburg Virginia USA; ^2^ Center for Depression, Anxiety, and Stress Research McLean Hospital Belmont Massachusetts USA; ^3^ Department of Psychiatry Harvard Medical School Boston Massachusetts USA; ^4^ Eating Disorders Clinical and Research Program, Department of Psychiatry Massachusetts General Hospital Boston Massachusetts USA; ^5^ Mass General Brigham Multidisciplinary Eating Disorders Research Collaborative Mass General Brigham Boston Massachusetts USA; ^6^ Psychiatry Neuroimaging Laboratory, Department of Psychiatry, Brigham and Women's Hospital Harvard Medical School Boston Massachusetts USA; ^7^ Neuroendocrine Unit, Department of Medicine Massachusetts General Hospital and Harvard Medical School Boston Massachusetts USA; ^8^ Kaiser Permanente Bernard J. Tyson School of Medicine Pasadena California USA; ^9^ Department of Neuroscience Harvard University Cambridge Massachusetts USA; ^10^ Division of Women's Mental Health McLean Hospital Belmont Massachusetts USA; ^11^ Department of Epidemiology Harvard T.H. Chan School of Public Health Boston Massachusetts USA; ^12^ Klarman Eating Disorders Center McLean Hospital Belmont Massachusetts USA; ^13^ Department of Medicine Harvard Medical School Boston Massachusetts USA; ^14^ McLean Imaging Center McLean Hospital Belmont Massachusetts USA; ^15^ Department of Psychiatry and Behavioral Sciences, Weill Institute for Neurosciences University of California San Francisco San Francisco California USA; ^16^ Division of Pediatric Endocrinology University of Virginia Charlottesville Virginia USA; ^17^ Division of Child and Adolescent Psychiatry, AMC Department of Psychiatry Massachusetts General Brigham Boston Massachusetts USA

**Keywords:** anorexia nervosa, cognitive flexibility, fMRI, restrictive eating disorders, task‐switching

## Abstract

**Objective:**

Restrictive eating disorders (EDs), including anorexia nervosa (AN) and atypical AN (Atyp‐AN), are often associated with cognitive rigidity that can impede treatment. The dorsolateral prefrontal cortex (dlPFC) plays a central role in cognitive control, but it remains unclear whether its activation during cognitive flexibility will differ across restrictive ED presentations.

**Method:**

Eighty‐seven females with restrictive EDs (aged 14–35) (AN: *n* = 31; atyp‐AN with history of AN [hx‐AN]: *n* = 33; atyp‐AN without history of AN [Atyp‐AN]: *n* = 23) completed a task‐switching paradigm during functional magnetic resonance imaging. We examined dlPFC activation during sustained (task‐switching vs. completing a single task) and transient control (switching between task rules vs. repeating the same rule), testing for group differences and symptom associations.

**Results:**

The AN group showed a greater difference in left dlPFC activation during task switching vs. the single task compared to the hx‐AN and Atyp‐AN groups, driven by reduced activation during the single task condition. All groups showed similar increases in dlPFC activation during task switching and no differences in task performance. Across all participants, higher dlPFC activation during task switching vs. single task was associated with greater restraint symptoms.

**Discussion:**

These novel findings identified shared versus unique neural features of sustained and transient control across restrictive ED groups. Heightened dlPFC activation during transient control associated with restraint may represent a transdiagnostic feature shared across restrictive EDs. Alternatively, reduced dlPFC activation during the low‐demand, single‐task condition in those with typical, but not atypical AN, may reflect a difference in sustained control, with implications for tailoring interventions to distinct restrictive ED presentations.

1

Summary

- Restrictive eating disorders, including anorexia nervosa (AN) and atypical AN, are often characterized by cognitive rigidity, but it is not clear whether neural activation during cognitive flexibility differs across restrictive eating disorder presentations.
- We compared neural activation during a task of cognitive flexibility across young women with AN, atypical AN with a history of AN, and atypical AN with no history of AN to determine shared versus unique neural and cognitive features of these restrictive eating disorder presentations.
- While all restrictive eating disorder groups showed similar increases in dorsolateral prefrontal cortex activation during task‐switching, the AN group demonstrated reduced dorsolateral prefrontal cortex activation during the low‐demand, single‐task condition, reflecting an alteration in sustained cognitive control unique to young women with AN.
- Interventions tailored to young women with AN may target sustained cognitive control, whereas task‐switching may be a transdiagnostic target for intervention across restrictive eating disorders.

## 2 Background

Restrictive eating disorders (EDs), including anorexia nervosa (AN) and atypical AN (Atyp‐AN), are serious psychiatric illnesses characterized by dietary restriction, intense fear of weight gain, and body image disturbance (APA 2022). While AN is defined by significantly low body weight, individuals with Atyp‐AN exhibit similar psychological symptoms and often experience significant weight loss yet maintain a weight within or above the normal range. Despite this distinction, both presentations are associated with high levels of clinical impairment, prolonged illness duration, and poor treatment response (Johnson‐Munguia et al. 2024; Walsh et al. 2023). These disorders often begin in adolescence and can follow a chronic, relapsing course. Moreover, restrictive EDs carry among the highest risks of premature mortality among psychiatric disorders (Arcelus et al. 2011; Keshaviah et al. 2014).

The clinical application of the Atyp‐AN diagnosis remains challenging, as it encompasses a heterogeneous group that may include individuals with higher‐weight AN (i.e., do not reach low weight in spite of weight loss and restriction), prodromal AN (i.e., are losing weight but have not yet reached the low weight threshold for AN diagnosis), or those in partial remission (i.e., history of low weight AN but now have persistent symptoms in the context of nonlow weight) (Eddy and Breithaupt 2023). This heterogeneity complicates efforts to define course, prognosis, and treatment needs. While efforts are ongoing to develop effective treatments for all restrictive ED presentations, available evidence‐based treatments for AN and Atyp‐AN are few, and those available are only effective for roughly half of patients (Monteleone et al. 2022; Solmi et al. 2024). Identifying mechanisms that contribute to illness persistence and treatment nonresponse, particularly those that differentiate or unify diagnostic presentations, remains a critical goal of ED research (Phillipou et al. 2025).

One such mechanism that may contribute to illness maintenance and poor outcomes is cognitive inflexibility, which is a well‐documented trait across restrictive EDs (Lang et al. 2014; Miles et al. 2020; Wang et al. 2021). Cognitive flexibility refers to the ability to shift mental strategies in response to changing circumstances (Miyake et al. 2000; Monsell 2003; Uddin 2021). Individuals with restrictive EDs demonstrate impaired performance on tasks requiring flexible adaptation (e.g., Task‐Switching, Wisconsin Card Sorting Test) compared to healthy controls (HC) (Miles et al. 2020). Reduced cognitive flexibility may underlie rigid behavioral patterns such as persistent dieting or difficulty adapting to treatment goals that contribute to chronicity and relapse. Indeed, cognitive flexibility is a direct treatment target in cognitive remediation therapy for restrictive EDs (Tchanturia et al. 2014, 2017); thus, improving our understanding of its neural underpinnings may identify new opportunities for intervention across restrictive ED presentations.

Consistent with these neuropsychological findings, functional magnetic resonance imaging (fMRI) studies have identified alterations in blood‐oxygenation‐level‐dependent (BOLD) signal in key brain regions and neural circuitry supporting cognitive flexibility, including the dorsolateral prefrontal cortex (dlPFC) and frontoparietal control network, in AN compared to HC. In case–control studies, individuals with AN show hypoactivation of frontoparietal regions during cognitive flexibility tasks compared to HC (Castro‐Fornieles et al. 2019; Sato et al. 2013; Wonderlich et al. 2021; Zastrow et al. 2009). Some evidence also suggests a more complex pattern of neural activation in individuals with AN (vs. HC), characterized by hyperactivation of the dlPFC to task rule changes but lower BOLD response when maintaining the same rule (Lao‐Kaim et al. 2015).

However, these studies focus on those with typical AN compared to HC, leaving open critical questions about whether neural signatures of cognitive flexibility vary across diagnostic presentations of restrictive EDs. Specifically, it is unknown whether patterns of dlPFC activation differ among individuals with typical AN, those who have restored weight but remain symptomatic (history of AN [hx‐AN]), and those with Atyp‐AN without a history of low‐weight AN. For instance, some studies indicate poorer cognitive flexibility only in the acute AN illness stage (Berner et al. 2019), whereas other studies found no differences in cognitive flexibility performance between acute and recovered AN (Gura‐Solomon et al. 2024; Miles et al. 2020). Moreover, it remains unclear whether individuals with Atyp‐AN, who may never have met the low‐weight threshold, share similar neural activation patterns with those who have a history of AN as cognitive flexibility in these patients is not well‐studied. Identifying neural signatures of cognitive flexibility that are shared vs. unique to these diagnostic presentations could provide insight into the neurobiological underpinnings of illness maintenance and relapse risk and help refine treatment targets.

Furthermore, contemporary models of cognitive flexibility distinguish the ability to *sustain readiness to engage cognitive flexibility* from the ability to *execute momentary cognitive flexibility* when required (Braver 2012; Monsell 2003)—an important distinction that has not been considered in the study of cognitive flexibility in restrictive EDs. Purposefully designed task‐switching paradigms capture this distinction. Task‐switching tasks ask participants to categorize the same stimuli using two simple rules (Task 1 and Task 2; e.g., categorize letters as vowel vs. consonant and upper vs. lower case). In task‐switch blocks, the rules alternate, creating task repeat and switch trials. In single‐task blocks, only one rule is applied. From the observed performance, two indices can be calculated. *Mixing costs* quantify performance decrements during task‐switching relative to single‐trial blocks, reflecting keeping both rules active (i.e., being ready to switch). *Switch costs* capture performance costs on switch versus repeat trials within mixed blocks, reflecting the momentary adjustment to a rule change (Kiesel et al. 2010; Monsell 2003; Rogers and Monsell 1995). Studies in other neuropsychiatric populations highlight how these components can dissociate, with individuals exhibiting increased mixing costs with intact switch costs or the reverse (Kray and Lindenberger 2000; Schmitter‐Edgecombe and Sanders 2009; Wylie et al. 2010). Limiting inference to the switch‐repeat contrast risks missing difficulties in sustained readiness.

Thus, we investigated dlPFC activation using an fMRI Task‐Switching Paradigm that captures sustained and transient cognitive flexibility to determine shared versus unique neural and behavioral markers of restrictive EDs. We analyzed data from adolescent and young adult women with typical AN, a history of typical AN who were currently weight‐restored (hx‐AN), and Atyp‐AN with no history of low weight. All participants endorsed current restrictive eating and body image disturbance, meeting DSM‐5‐TR criteria B and C for AN, but only the AN group met the low‐weight criterion (criterion A) (APA 2022). Based on prior findings of altered frontoparietal activation in individuals with AN (Castro‐Fornieles et al. 2019; Sato et al. 2013; Wonderlich et al. 2021; Zastrow et al. 2009), we hypothesized that women with typical AN compared to women with a history of AN or Atyp‐AN would show greater differences in dlPFC activation (a) during the task‐switching versus single‐task blocks (isolating tonic engagement of the frontoparietal control network; Hypothesis 1) and (b) in trials switching from one task to another versus task repeat (transient reconfiguration processes; Hypothesis 2). As an additional exploratory aim, we tested relations between dlPFC activation contrasts that revealed group differences and severity of ED symptoms and task performance across the whole sample to probe whether dlPFC engagement tracks the dimensional intensity of restrictive ED independent of diagnostic status.

## 3 Method

### 3.1 Participants

Cross‐sectional data were available from 103 hypoestrogenic young women aged 14–35 years with EDs characterized by restrictive eating who had missed ≥ 3 periods in the last 6 months. These data are a part of an ongoing randomized controlled trial examining the role of estrogen replacement on cognitive flexibility and reward responsiveness (NCT03740204). Women were recruited from local treatment centers and through university and community postings online, in person, and social media advertisements (for additional details, see Breithaupt et al. 2025). Inclusion criteria were: females, 14–35 years old (ages selected to ensure females were postmenarchal and premenopausal); diagnoses of DSM‐5 AN, Atyp‐AN, other restricting‐type other specified feeding and eating disorder (OSFED) presentations; and hypoestrogenism (indicated by oligo‐amenorrhea or missing ≥ 3 periods in the last 6 months). Exclusion criteria were: active suicidal ideation; DSM‐5 binge‐eating disorder or OSFED subthreshold binge eating disorder; oligo‐amenorrhea caused by pregnancy, polycystic ovary syndrome, thyroid dysfunction, primary ovarian insufficiency, or hyperprolactinemia; medications containing estrogen ± progesterone; neurological or psychiatric disorders with psychotic features or active substance use disorders; lifetime history of seizure disorder or electro‐convulsive therapy; and contraindications to MRI or estrogen use. Participants on progestin‐releasing intrauterine devices were included if labs demonstrated low estradiol levels. Written informed consent was obtained from participants ≥ 18 years and written consent and assent were obtained from parents and participants < 18 years, respectively, as approved by the MassGeneral Brigham Institutional Review Board.

We administered the Eating Disorders Examination (EDE) clinical interview (Byrne et al. 2010; Cooper and Fairburn 1987), a clinical review of symptoms and history, and participants completed self‐report questionnaires. Individuals who met current criteria for AN were assigned to the AN group. Individuals who currently met criteria for AN except for the low‐weight threshold (i.e., criteria B and C) were assigned to the hx‐AN group if they had a history of meeting full criteria for low‐weight AN, or they were assigned to the Atyp‐AN group if they had no low‐weight AN history (there was no required degree of weight suppression or loss). Four participants did not meet the criteria for any of the groups (i.e., bulimia nervosa) and were excluded from analyses.

Of the 99 participants with MRI data, 6 participants were excluded due to poor task‐switching performance (≥ 30% error during the single‐task condition), 5 participants were excluded due to not meeting fMRI data quality assurance (> 10% of trials with framewise displacement [FD] > 1 mm and DVARS > 1.5 or > 5 trials with > 3 mm FD), and one participant was excluded due to the presence of an incidental MRI finding. The final sample included 87 participants presenting with DSM‐5‐TR AN (*n* = 31), hx‐AN (*n* = 33), and Atyp‐AN (*n* = 23).

### 3.2 ED Symptoms

Participants completed the EDE semi‐structured clinical interview (Byrne et al. 2010; Cooper and Fairburn 1987) and mental‐health questionnaires assessing ED symptoms. Core ED features of interest included restraint, measured with the EDE Dietary Restraint (Black and Terence Wilson 1996); restriction measured with the EPSI Dietary Restriction scale (Forbush et al. 2013); and drive for thinness assessed by the Eating Disorders Inventory (EDI‐3) Drive for Thinness scale (Garner 2004) (see Supporting Information S1).

### 3.3 Task‐Switching fMRI Paradigm

**FIGURE 1:**
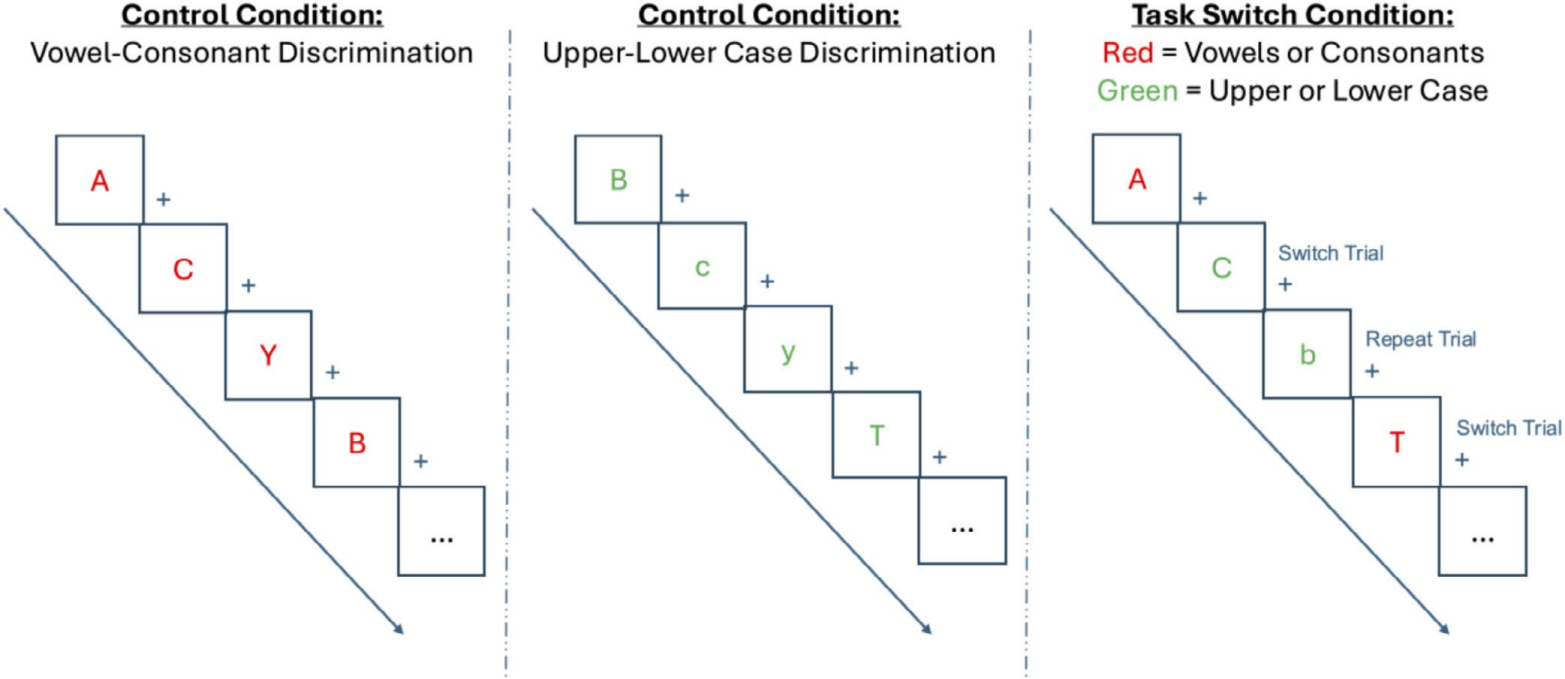
Task‐switching fMRI paradigm. Depiction of the task‐switching paradigm adapted from Girard et al. (2017). Participants were asked to respond manually using button presses to visually presented letters in three task conditions: vowel‐consonant discrimination (left panel); upper‐lower case discrimination (middle panel); and task‐switching (right panel). The vowel‐consonant and upper‐lower case discrimination conditions were single task control conditions in which participants had to choose between vowels and consonants or upper and lower case letters. In the task‐switching condition, participants randomly switched between these discrimination tasks with the color indicating the task (red = vowel‐consonant discrimination; green = upper‐lower case discrimination). Switch trials were defined as trials in which participants alternated task instructions from the previous task. Repeat trials were defined as trials in which participants repeated the same task as the prior trial. In the three conditions, the stimulus duration was 1500 ms and the inter‐trial interval (ITI; fixation cross) was jittered between 500 and 4500 ms (shown as “+” in the figure).

Cognitive flexibility was assessed with a cued letter task‐switching paradigm during fMRI (adapted from Girard et al. 2017; Figure 1). In each trial, participants categorized a letter as either vowel versus consonant (Task 1) or upper versus lower case (Task 2). The letter color determined the task. In the task‐switch condition, Tasks 1 and 2 were mixed in the same block, creating task repeat and switch trials. In the single‐task control condition, blocks of only Task 1 or Task 2 were presented. Percent error and reaction time (RT) were computed. We examined two effects: (1) *mixing costs* as the performance difference between the task‐switching condition (all trials) and the single‐task condition, indexing performance decrements due to sustained readiness to alternate; and (2) *switch costs* as the performance difference between task switch and task repeat trials within the task‐switch condition, indexing costs of momentary rule change (higher values = poorer cognitive flexibility) (see Supporting Information S1).

### 3.4 Delis‐Kaplan Executive Functioning System (D‐KEFS)

Participants completed the D‐KEFS Color Word Interference Test outside the scanner as a measure of executive functioning (Delis et al. 2001). We report t‐scores on the Inhibition/Switching condition to characterize the groups on an external cognitive flexibility measure.

### 3.5 MRI Acquisition and Preprocessing

Data were acquired on a 3T Prisma scanner with a 64‐channel head–neck coil at the McLean Imaging Center. Detailed information on MRI acquisition, preprocessing, and quality assurance is described elsewhere (Breithaupt et al. 2025) and in the Supplement. Briefly, high‐resolution anatomical data were acquired using a T1 magnetized‐prepared rapid acquisition with gradient echo (MPRAGE) imaging sequence with the following acquisition parameters: 176 sagittal slices, repetition (TR) = 2530 ms, echo times (TE) = 1.69, 3.55, 5.41, 7.27 ms, 7° flip angle, 1 × 1 × 1 mm voxels, field of view = 256 mm. Whole‐brain gradient echo T2*‐weighted echo planar images (EPI) were acquired using the U. Minnesota multi‐band BOLD EPI sequence with the following parameters: TR/TE = 2000/30 ms; 80° flip angle, voxels 1.5 × 1.5 × 1.5 mm, field of view = 204 mm, multiband factor 3. Data were preprocessed using fMRIPrep 20.0.1 (Esteban et al. 2019) including distortion, slice time, and motion corrections, denoising using ICA‐AROMA, and smoothing and normalization to MNI1526Asym space.

### 3.6 fMRI Analyses

Statistical analyses of single‐subject fMRI data were implemented using a general linear model with regressors corresponding to “vowel‐consonant discrimination” (single‐task), “upper‐lower case discrimination” (single‐task), task‐switch “repeat” and task‐switch “switch” (within task‐switch block), incorrect (i.e., trials with incorrect answers), and no response (i.e., trials for which the subject did not respond) trials in SPM12. Each event was constructed with a hemodynamic response function, modeled using a gamma function, and convolved with onset times of events and stimulus duration. Contrast maps of (a) task‐switch vs. single‐task (average of vowel‐consonant and upper‐lower case discrimination blocks; Girard et al. 2017) conditions were constructed as an index of *sustained cognitive flexibility*, and (b) switch vs. repeat trials within the task‐switch condition as a measure of *transient cognitive flexibility*. We examined group differences in brain activation using region‐of‐interest (ROI) and whole‐brain approaches.

### 3.7 ROI Analyses

The contrast maps were used to test differences in neural activation of bilateral dlPFC ROIs across the three restrictive ED groups during task‐switch vs. single‐task conditions and switch vs. repeat trials within the task‐switching condition. dlPFC ROIs were created from the FSL Harvard‐Oxford Cortical/Subcortical Atlas using a 60% probability threshold. fMRI BOLD estimates within the left and right dlPFC ROIs were extracted from the contrast maps. Four repeated‐measures analyses of variances (ANOVAs) were conducted to examine group differences in the left and right dlPFC ROIs during the task‐switch vs. single‐task condition and switch vs. repeat trials. The repeated‐measures ANOVAs had one between‐subjects factor (AN, hx‐AN, Atyp‐AN groups) and one within‐subjects factor (condition/trial type). For any significant task condition × group interactions, post hoc independent‐samples *t*‐tests were conducted comparing dlPFC activation during each condition or trial type between each pair of groups. To address the exploratory aim, multiple regression analyses were conducted to examine associations between dlPFC BOLD estimates and task performance and ED symptoms. All analyses included age as a covariate. We employed a false discovery rate (FDR) procedure (Benjamini and Hochberg 1995) (*q* < 0.05) to control for multiple testing within each set of tests.

We also conducted sensitivity analyses to test the robustness of our ROI findings. First, we additionally controlled for mean FD as a measure of head motion during the paradigm. Second, we additionally controlled for depression and anxiety symptoms, measured with the Beck Depression Inventory (BDI‐II) (Beck et al. 2011) and State‐Trait Anxiety Inventory‐Trait scale (STAI‐T) (Spielberger et al. 1999), respectively, to ensure our findings were not driven by co‐occurring internalizing symptoms.

### 3.8 Whole‐Brain Analyses

In addition to the hypothesis‐driven ROI analyses, we conducted whole‐brain exploratory analyses. First, we examined the main effects of the task‐switch vs. single‐task condition and switch vs. repeat trials across all participants. Second, to determine whether there were group differences in the broader cognitive‐control network, we conducted one‐way ANOVAs to examine diagnostic group differences in neural activation during the task‐switch vs. single‐task condition and switch vs. repeat trials controlling for age. A significance threshold of *p* < 0.05 family‐wise error (FWE) cluster correction with an initial voxel forming threshold of uncorrected *p* < 0.005 was employed. For any significant task condition × group interactions, we ran post hoc independent‐samples t‐tests comparing neural activation during each condition or trial type between each pair of groups.

## 4 Results

### 4.1 Participant Characteristics and Symptom Profiles

Descriptive statistics for the full sample and each restrictive ED group are summarized in Table 1. Groups did not significantly differ on any demographic or task performance variable. The AN group had a lower BMI and reported higher EPSI Restriction scores relative to the hx‐AN and Atyp‐AN groups. Across the full sample, higher error rate mixing costs were associated with higher EDE Dietary Restraint (*r* = −0.314, *p* = 0.002) and EPSI Restriction (*r* = 0.264, *p* = 0.012) but were not associated with other symptom measures (see Table S1 for bivariate correlations among all variables).

**TABLE 1:** Descriptive statistics of study variables for the overall included sample and for each diagnostic group.

	Overall sample (*n* = 87)	AN (*n* = 31)	Hx‐AN (*n* = 33)	Atyp‐AN (*n* = 23)	*F*/*X* ^2^	*p*
	Mean (SD)	Mean (SD)	Mean (SD)	Mean (SD)		
Demographic variables						
Age (years)	21.91 (4.14)	22.07 (4.08)	22.38 (4.23)	21.03 (4.16)	0.75	0.475
Race, No. (%)					6.67	0.352
White	69 (79.3)	25 (80.6)	24 (72.7)	20 (87.0)		
Asian	12 (13.8)	3 (9.7)	7 (21.2)	2 (8.7)		
Other	2 (2.3)	0 (0)	1 (3.0)	1 (4.3)		
More than one race	4 (4.6)	3 (9.7)	1 (3.0)	0 (0)		
Mean FD	0.128 (0.06)	0.110 (0.04)	0.131 (0.06)	0.146 (0.07)	2.80	0.067
BMI (kg/m^2^)	19.74 (2.27)	17.64 (1.58)	20.35 (1.10)	21.70 (2.01)	48.83	**< 0.001** ^a,b,c^
Clinical symptom measures						
EDE Dietary Restraint	2.76 (1.55)	3.16 (1.49)	2.44 (1.66)	2.64 (1.43)	1.80	0.172
EPSI Restriction	9.68 (5.02)	11.65 (4.18)	9.07 (5.19)	7.78 (5.12)	4.60	**0.013** ^b^
EDI Drive For Thinness	17.10 (7.34)	18.06 (7.32)	16.45 (7.55)	16.74 (7.25)	0.42	0.660
BDI	17.67 (12.01)	20.42 (10.98)	16.82 (13.42)	14.95 (11.17)	1.45	0.241
STAI‐T	51.25 (11.55)	54.23 (10.08)	49.64 (12.53)	49.57 (11.61)	1.62	0.204
Task performance measures						
Error rate single task (%)	6.53 (4.83)	6.16 (5.40)	7.13 (5.05)	6.18 (3.66)	0.41	0.667
RT single task (ms)	621.72 (69.91)	625.70 (70.56)	622.17 (70.09)	615.70 (71.51)	0.13	0.875
Error rate task‐switching (%)	18.81 (8.50)	19.78 (10.17)	18.86 (7.51)	17.43 (7.51)	0.50	0.607
RT task‐switching (ms)	820.51 (115.34)	835.85 (115.38)	815.52 (106.15)	807.00 (130.01)	0.46	0.635
Error rate mixed costs (%)	12.28 (7.99)	13.63 (10.41)	11.73 (6.13)	11.25 (6.56)	0.71	0.497
RT mixed costs (ms)	198.79 (92.32)	210.16 (96.64)	193.34 (75.46)	191.30 (109.60)	0.36	0.697
Error switch costs (%)	5.07 (9.27)	5.10 (10.61)	6.37 (8.63)	3.17 (8.22)	0.80	0.452
RT switch costs (ms)	94.84 (63.80)	114.98 (59.27)	88.01 (59.47)	77.50 (70.85)	2.68	0.074
D‐KEFS Color Word Inhibition/Switching	52.10 (8.64)	49.19 (10.51)	54.27 (6.21)	52.91 (8.10)	3.04	0.053

*Note*: Groups were compared with ANOVAs (for continuous measures) or chi‐squared tests (for categorical measures). *p*‐values < 0.05 are shown in bold. Superscripts on the significant *p*‐values denote which groups were significantly different from each other as indicated by Tukey's HSD: (a) AN vs. hx‐AN; (b) AN vs. Atyp‐AN; (c) hx‐AN vs. Atyp‐AN.

Abbreviations: AN = Anorexia Nervosa; BDI = Beck Depression Inventory; BMI = Body Mass Index; D‐KEFS = Delis‐Kaplan Executive Function System; EDE = Eating Disorders Examination; EDI = Eating Disorders Inventory; FD = framewise displacement; RT = reaction time; STAI‐Trait = State‐Trait Anxiety Inventory‐Trait.

### 4.2 Whole‐Brain Neural Activation During Task‐Switching

**FIGURE 2:**
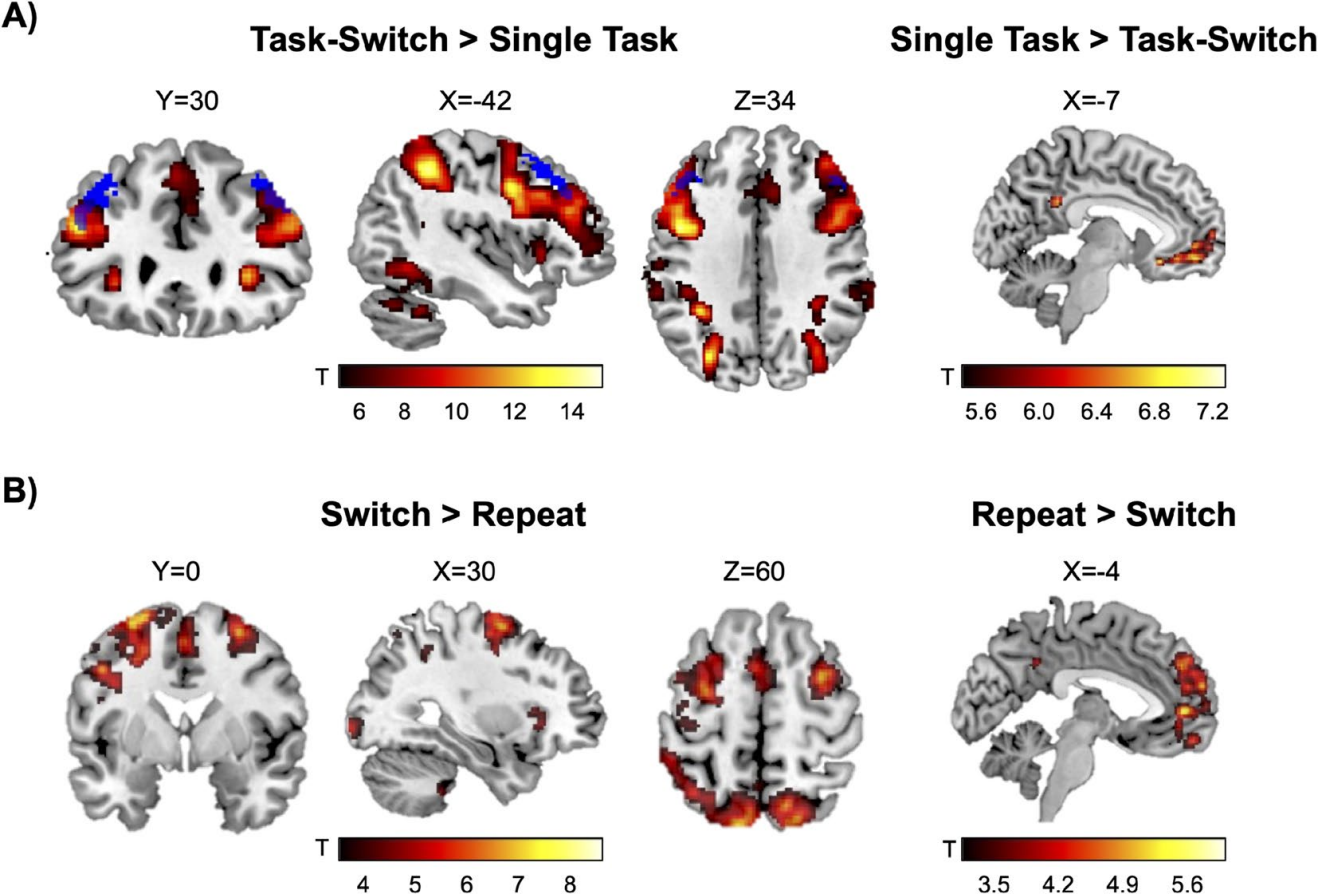
Main effects of task‐switching. Statistical parametric maps from whole‐brain analyses are shown to illustrate voxels exhibiting significant main effects of (A) task‐switching > single task (left panel; reverse contrast on right panel) and (B) switch > repeat trials during the task‐switching condition (left panel; reverse contrast on right panel). The dlPFC ROI is shown in blue and overlaid onto A (left panel). During both task‐switch > single task and switch > repeat contrasts, there was greater activation in expected cognitive control regions including within the middle and superior frontal gyri, parietal, lateral occipital, and posterior cerebellar regions (see Table S2 for complete list of significant clusters). The panels on the right (A and B) show activation within default mode network regions during the reverse contrasts (i.e., single task > task‐switch and repeat > switch), consistent with the expected task‐driven engagement of executive networks. Colorbars reflect t‐scores. A significance threshold of *p* < 0.05 family‐wise error (FWE) cluster correction was employed for the task‐switch vs. single task contrast. An initial voxel forming threshold of uncorrected *p* < 0.001 for the switch vs. repeat trials was employed with a significance threshold of *p* < 0.05 FWE cluster correction.

Across the full sample, during task‐switch vs. single‐task, there was greater activation in expected cognitive control regions including the middle frontal gyrus, superior parietal lobule, supramarginal gyrus, lateral occipital cortex, temporal gyrus, intracalcarine cortex, thalamus, caudate, and posterior cerebellum (Figure 2A), consistent with prior task‐switching fMRI studies (Girard et al. 2017; Zastrow et al. 2009). During switch vs. repeat trials within the task‐switching condition, there also was greater activation in cognitive control regions including the middle and superior frontal gyri (Figure 2B and Table S2). Decreased activation in default mode network regions was found during the task‐switch vs. single‐task and switch vs. repeat trials, consistent with task‐driven engagement of executive networks.

### 4.3 Group Differences in ROI Analyses

**FIGURE 3:**
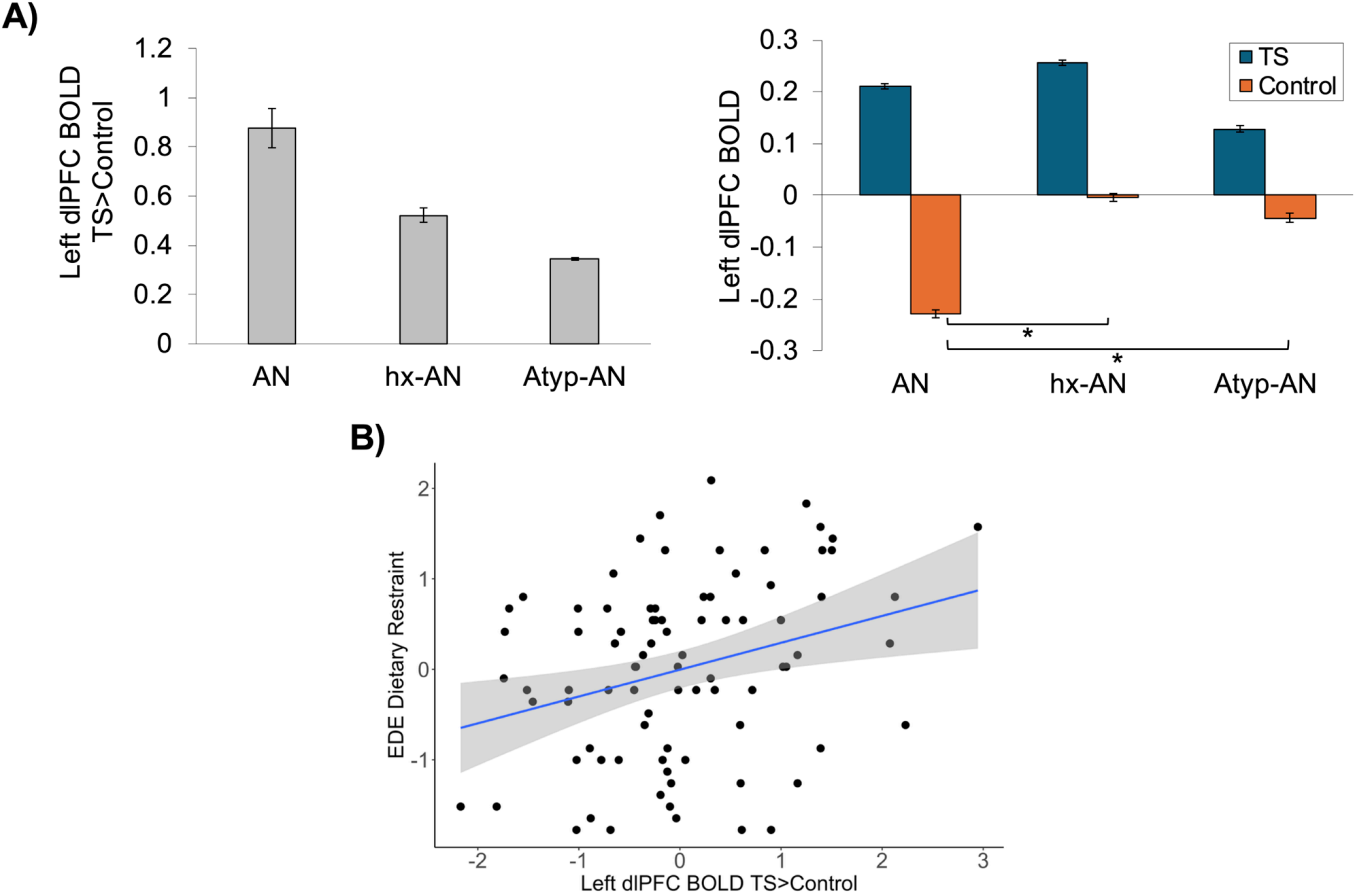
Diagnostic group differences in left dlPFC activation during task‐switching vs. single task and associations with restraint. (A) Bar charts are shown demonstrating significant diagnostic group differences in left dlPFC activation during task‐switch vs. single task conditions (left panel) and the significant task condition × diagnostic group interaction (right panel). The AN group demonstrated a significantly greater difference in left dlPFC activation during task‐switch vs. single task than the hx‐AN and Atyp‐AN groups. The task condition × diagnostic group interaction was driven by lower left dlPFC activation during the single‐task condition in the AN group compared to the hx‐AN and Atyp‐AN groups. Between‐group standard error bars ±2 are shown. (B) Post hoc partial regression plots are shown of the significant relations between left dlPFC BOLD during task‐switch vs. single‐task conditions and EDE Dietary Restraint controlling for age. **p < 0.01; *p < 0.05.

We compared activation in bilateral dlPFC ROIs during the task‐switch vs. single‐task condition and switch vs. repeat trials across the restrictive ED groups. A task condition × group interaction emerged in the left dlPFC (*F*(2,83) = 4.22, *p* = 0.018, *ηp* ^2^ = 0.092), but not in the right dlPFC ROI (*p* = 0.694) for the task‐switch vs. single‐task condition (Figure 3A,B; Figure S1). This interaction effect remained robust to the inclusion of mean FD, BDI‐II, and STAI‐T as additional covariates. Post hoc *t*‐tests demonstrated this interaction was driven by lower left dlPFC activation during the single‐task condition in the AN group compared to the hx‐AN [*t*(62) = −2.676, *p* = 0.010] and Atyp‐AN groups [*t*(52) = −2.230, *p* = 0.030]. There were no task condition × group interactions in the dlPFC during switch vs. repeat trials (*ps* > 0.300). These findings suggest that the group differences were driven by the reduced single‐task condition dlPFC activation in those with typical AN, rather than by greater cognitive flexibility demands during task‐switching.

### 4.4 Associations Between dlPFC Activation and Task Performance and ED Symptoms

To address our exploratory aim, follow‐up associations of the left dlPFC BOLD estimates during the task‐switch vs. single‐task condition with task performance and symptom measures were conducted across all participants (Table 2). Greater left dlPFC BOLD during task‐switch vs. single‐task was associated with higher EDE Dietary Restraint (*β* = 0.302, 95% CI [0.094, 0.509], *p* = 0.005) after FDR correction for the 10 tests. These relations were robust to the inclusion of mean FD, BDI‐II, and STAI‐T as additional covariates. Left dlPFC BOLD also was significantly positively associated with the EPSI Cognitive Restraint scale (see Supporting Information S1), suggesting these findings were robust to different measures of restraint. Left dlPFC activation during the task‐switch vs. single‐task condition was unrelated to EDI Drive for Thinness, EPSI Restriction, or task performance.

**TABLE 2:** Associations between left dlPFC activation during task‐switching vs. single task and symptom and task performance across all participants.

	Std. B	95% CI	*p*
BMI	−0.065	[−0.283, 0.152]	0.552
EDE dietary restraint	0.302	[0.094, 0.509]	**0.005**
EPSI restriction	0.076	[−0.145, 0.297]	0.494
EDI drive for thinness	0.187	[−0.025, 0.400]	0.084
RT mixed costs (ms)	0.170	[−0.039, 0.379]	0.110
Error rate mixed costs (%)	0.202	[−0.011, 0.414]	0.063
RT single task (ms)	0.087	[−0.121, 0.295]	0.407
Error rate single task (%)	−0.040	[−0.243, 0.164]	0.699
RT TS (ms)	0.189	[−0.024, 0.402]	0.082
Error rate TS (%)	0.167	[−0.043, 0.377]	0.118

*Note*: Standardized estimates and 95% confidence intervals (CIs) are shown from separate regression models of left dlPFC activation during task‐switch vs. single‐task conditions relating to each of the 10 symptom and task performance variables in the first column. Age was included as a covariate. *p*‐values are unadjusted; *p*‐values that survived FDR correction for the 10 tests (*q* < 0.05) are indicated in bold.

Abbreviations: BDI = Beck Depression Inventory; BMI = Body Mass Index; EDE = Eating Disorders Examination; EDI = Eating Disorders Inventory; RT = reaction time; STAI‐T = State‐Trait Anxiety Inventory‐Trait.

To determine whether the relation between EDE Restraint and left dlPFC activation was driven by activation during the task‐switch or single‐task condition, we tested this relation separately by condition (Table S3). Although nonsignificant, higher EDE Restraint was associated with greater left dlPFC BOLD during the task‐switch (*β* = 0.191, 95% CI [−0.022, 0.403], *p* = 0.078) but not the single‐task condition (*β* = −0.086, 95% CI [−0.302, 0.130], *p* = 0.430). These relations were significantly different from each other according to Meng's test (Meng et al. 1992): *z* = −2.56, *p* = 0.01. Additionally, within‐group regressions determined that the association between EDE Restraint and left dlPFC BOLD across all participants was driven by those with AN (*β* = 0.351, 95% CI [−0.009, 0.582], *p* = 0.057) and hx‐AN (*β* = 0.344, 95% CI [−0.019, 0.999], *p* = 0.058), but not Atyp‐AN (*β* = 0.055, 95% CI [−0.402, 0.513], *p* = 0.803) and remained after controlling for BMI.

### 4.5 Exploratory Whole‐Brain Analyses of Group Differences

Whole‐brain one‐way ANOVA revealed task condition × group interactions in the left superior medial frontal cortex, partially overlapping with the dlPFC ROI, and left cerebellar Crus I/II and right medial orbitofrontal cortex during the task‐switch vs. single‐task condition (Table S4). There were no task condition × group interactions for switch vs. repeat trials. We extracted BOLD estimates from these three clusters to conduct post hoc t‐tests comparing neural activation between each pair of groups for the task‐switch and single‐task conditions. Similar to the ROI results, these interactions were driven by differences in BOLD response during the single‐task condition between the AN and hx‐AN groups (Figure S2 and Supporting Information S1).

## 5 Discussion

We examined whether dlPFC activation during a cognitive flexibility fMRI task capturing both sustained (mixing) and transient (switch) control demands differentiates among restrictive ED diagnostic presentations. Among 87 young women with typical AN, weight‐restored AN (hx‐AN), or Atyp‐AN, we found significant group differences in left dlPFC activation when switching between tasks compared to performing a single task (mixing costs), independent of age, head motion, and comorbid internalizing symptoms. Specifically, young women with typical AN demonstrated a greater difference in left dlPFC activation between task‐switch and single‐task conditions than the hx‐AN and Atyp‐AN groups, but there were no group differences in behavioral task performance.

Contrary to our hypothesis, the dlPFC group effect was driven by reduced activation in the typical AN group during the single‐task baseline, not by exaggerated recruitment during task‐switching. Whole‐brain exploratory analyses further supported these findings, identifying additional group differences in fronto‐cerebellar and prefrontal regions, again driven by reduced activation during the single‐task condition in the typical AN group. This pattern implies a potential deficit in tonic task engagement/sustained control rather than in the transient reconfiguration processes traditionally indexed by switch trials. This distinguishing feature in those with typical AN who were acutely underweight versus hx‐AN and Atyp‐AN suggests that this single‐task under‐engagement may be state‐related, potentially linked to malnutrition, low energy availability, and/or reduced motivation. However, given that the typical AN group also reported greater severity of restrictive eating pathology, we cannot rule out the possibility that these effects were driven by ED symptom severity rather than low‐weight status.

Alternatively, this lower activation during the single‐task baseline in the typical AN versus hx‐AN and Atyp‐AN groups could reflect neural efficiency rather than a deficit, particularly given the absence of behavioral performance differences and a healthy control group. However, prior case–control studies (e.g., Lao‐Kaim et al. 2015) have found reduced neural response during repetitive rule maintenance in typical AN compared to HCs, which supports the interpretation that reduced single‐task recruitment reflects under‐engagement rather than efficiency. Further supporting this interpretation, our dimensional analyses revealed that greater left dlPFC response during task‐switching relative to the single‐task baseline was associated with greater ED restraint symptoms. Nevertheless, future studies including healthy controls are needed to determine whether reduced dlPFC activation during sustained control represents a deficit or efficiency in typical AN.

All groups showed similar increases in left dlPFC activation during transient switching between tasks versus task repeats (Hypothesis 2). This finding suggests that dlPFC engagement during transient task‐switching is shared across restrictive ED presentations. This pattern aligns with prior case–control studies which found elevated dlPFC activation during task‐switching and attenuated neural response during repetitive rule maintenance in individuals with typical AN compared to HC (e.g., Lao‐Kaim et al. 2015). Our results extend this research in two ways: (1) we identified specific dlPFC alterations in sustained control during the single‐task baseline rather than alterations in transient reconfiguration processing/reactive control during the higher‐demand task‐switching; and (2) we determined that this alteration is most pronounced in individuals with acute low‐weight AN. Together, these results underscore the value of parsing cognitive flexibility by distinguishing sustained and transient components across restrictive ED groups.

Across the full sample, exploratory dimensional analysis revealed that higher left dlPFC activation during task‐switching (relative to single task) was associated with higher restraint, but not restriction, drive for thinness, or BMI, indicating dlPFC engagement is more closely tied to goal‐directed, cognitive aspects of restrictive behavior rather than to general ED psychopathology. When further probing these associations, we found that the overall positive relation between restraint and left dlPFC activation during the task‐switching versus single‐task condition was driven by greater activation during task‐switching rather than the single‐task blocks. Further, our follow‐up results suggested that this overall positive association between restraint and left dlPFC activation was most prominent in those with typical AN or a hx‐AN rather than those with Atyp‐AN, suggesting this relation may be driven by lifetime low‐weight status. However, as these associations were uncovered post hoc, they should be treated as hypothesis‐generating for future studies.

### 5.1 Limitations and Future Directions

Our study had the following limitations. First, as this study was designed to contrast restrictive ED groups, we did not include a HC group, limiting conclusions about whether dlPFC activation during cognitive flexibility is abnormal across the restrictive ED presentations. Moreover, the Atyp‐AN group may include individuals who are weight suppressed relative to their growth trajectory, complicating the interpretation of whether observed neural differences reflect acute physiological state versus transdiagnostic characteristics. Importantly, there is little consensus on how to best operationalize weight suppression (e.g., absolute vs. relative reduction from premorbid weight) or the timeframe over which weight suppression exerts physiological and neural effects. Without data characterizing weight suppression in this sample, we are unable to examine the impact of weight suppression independent of low weight. Second, our sample included predominantly White young women who were hypoestrogenic; thus, our findings may not generalize to nonhypoestrogenic or older women, those from other racial/ethnic backgrounds, or males. Furthermore, restrictive eating is a pattern shared across many EDs and whether our findings generalize to other ED presentations warrants future study. Third, our cross‐sectional design relied on retrospective clinical data to define diagnostic subgroups, which limits causal inference about illness progression. We did not collect information on the length of time since weight restoration occurred in the hx‐AN group. Future longitudinal studies are needed to determine whether dlPFC activation normalizes with weight restoration and sustained recovery.

Fourth, because all groups were symptomatic, we cannot determine whether observed similarities reflect enduring traits or current illness expression. Disentangling state‐ versus trait‐related effects in AN is inherently complex and will likely remain a challenge for the field. The heterogeneity of Atyp‐AN and absence of standardized definitions of weight suppression further complicate these efforts. Nevertheless, these challenges underscore the importance of continuing to pursue this line of inquiry. Parsing state and trait contributions is essential for staging illness and tailoring interventions; our results identifying neural alterations that are shared versus unique to restrictive ED groups is the first step towards this goal. For example, this line of inquiry could identify neural alterations that normalize with acute weight restoration versus those that persist, which may reflect more enduring vulnerabilities requiring targeted remediation. Future work should continue to refine approaches for delineating state and trait effects to inform treatment strategies that address both acute physiological states and longer‐term trait liabilities.

## 6 Conclusions

We found significant differences in neural activation of the dlPFC that distinguished between typical, weight‐restored, and nonunderweight restrictive ED presentations, and transdiagnostic associations with ED restraint. Contrary to our hypothesis, dlPFC differences across groups were driven by under‐engagement during the single‐task baseline in low‐weight AN, rather than heightened recruitment during switching. This pattern implicates an alteration in tonic/sustained control, while neural activation for switch vs. repeat trials indexing transient task set reconfiguration/reactive control was shared across groups. The observed pattern highlights a possible dissociation between alterations in sustained and transient control as a distinguishing feature among these restrictive ED subgroups. This work underscores the importance of examining shared versus unique neural and cognitive features of these distinct restrictive ED presentations. Future research should include a healthy control group to determine whether these alterations represent abnormalities that could be targeted in interventions tailored to specific restrictive ED presentations.

## Author Contributions

**Adrienne L. Romer:** writing – original draft, writing – review and editing, conceptualization, formal analysis, supervision, methodology, data curation, visualization. **Lauren Breithaupt:** writing – original draft, writing – review and editing, supervision, project administration, methodology, investigation, data curation, conceptualization. **Meghan Slattery:** writing – review and editing, project administration, data curation. **Felicia Petterway:** writing – review and editing, project administration, data curation. **Lauren Lindman:** writing – review and editing, project administration, data curation. **Jason Scott:** writing – review and editing, project administration, data curation. **Meghan Lauze:** writing – review and editing, project administration, data curation. **Mia Cravitz:** writing – review and editing, project administration, data curation. **Zara Poon:** writing – review and editing, project administration, data curation. **Siddarth Seenivasa:** writing – review and editing, supervision, methodology, funding acquisition, data curation. **Sarah Naticchia:** writing – review and editing, project administration. **Kristin N. Javaras:** writing – review and editing, supervision, data curation. **David Alperovitz:** writing – review and editing, supervision, resources, data curation. **Jennifer J. Thomas:** writing – review and editing, supervision, investigation. **Elizabeth A. Lawson:** writing – review and editing, supervision, investigation. **Diego A. Pizzagalli:** writing – review and editing, resources, methodology, investigation, conceptualization. **Franziska Plessow:** writing – review and editing, writing – original draft, conceptualization, supervision, methodology, funding acquisition, data curation. **Poornima Kumar:** writing – review and editing, writing – original draft, conceptualization, supervision, methodology, data curation, formal analysis, investigation, visualization. **Madhusmita Misra:** writing – review and editing, supervision, resources, methodology, investigation, funding acquisition, conceptualization. **Kamryn T. Eddy:** writing – review and editing, writing – original draft, supervision, resources, methodology, investigation, funding acquisition, data curation, conceptualization.

## Conflicts of Interest

Over the past 3 years, D.A.P. has received consulting fees from Arrowhead Pharmaceuticals, Boehringer Ingelheim, Compass Pathways, Engrail Therapeutics, Neumora Therapeutics, Neurocrine Biosciences, Neuroscience Software, and Takeda; he has received honoraria from the American Psychological Association, Psychonomic Society and Springer (for editorial work) and from Alkermes; he has received research funding from the BIRD Foundation, Brain and Behavior Research Foundation, Dana Foundation, DARPA, Millennium Pharmaceuticals, NIMH and Wellcome Leap MCPsych; he has received stock options from Ceretype Neuromedicine, Compass Pathways, Engrail Therapeutics, Neumora Therapeutics, and Neuroscience Software. E.A.L. receives grant support and research study drug from Tonix Pharmaceuticals and receives royalties from UpToDate. E.A.L. and/or immediate family members hold/recently held stock in Thermo Fisher Scientific, Zoetis, Danaher Corporation, Intuitive Surgical, Merck, West Pharmaceutical Services, Gilead Sciences, and Illumina. E.A.L. and F.P. are inventors on PCTUS2025/030536 entitled, “Oxytocin‐based therapeutics to improve cognitive control in individuals with attention deficit hyperactive disorder” filed on May 22, 2025. Over the past 3 years, K.N.J. owned equity shares in Sanofi and Centene Corporation, served on the Clinical Advisory Board for Beanbag Health, and received research funding from the National Institute of Diabetes and Digestive and Kidney Diseases and the Brain & Behavior Research Foundation. No funding from these entities was used to support the current work, and all views expressed are solely those of the authors. The authors declare no conflicts of interest.

## Supporting information


**Data S1:** eat24599‐sup‐0001‐Supinfo.docx.

## Data Availability

The data that support the findings of this study are openly available in the National Institute of Mental Health Data Archive (NDA) at https://doi.org/10.15154/3srm‐ca32, reference number 3148.

## References

[eat24599-bib-0001] APA . 2022. American Psychological Association: Diagnostic and Statistical Manual of Mental Disorders. 5th ed., Text Rev. ed. American Psychiatric Association.

[eat24599-bib-0002] Arcelus, J. , A. J. Mitchell , J. Wales , and S. Nielsen . 2011. “Mortality Rates in Patients With Anorexia Nervosa and Other Eating Disorders: A Meta‐Analysis of 36 Studies.” Archives of General Psychiatry 68, no. 7: 724–731. 10.1001/archgenpsychiatry.2011.74. 21727255

[eat24599-bib-0003] Beck, A. T. , R. A. Steer , and G. Brown . 2011. Beck Depression Inventory–II. APA PsycTests. 10.1037/t00742-000.

[eat24599-bib-0004] Benjamini, Y. , and Y. Hochberg . 1995. “Controlling the False Discovery Rate: A Practical and Powerful Approach to Multiple Testing.” Journal of the Royal Statistical Society, Series B: Statistical Methodology 57, no. 1: 289–300.

[eat24599-bib-0005] Berner, L. A. , E. M. Romero , E. E. Reilly , J. M. Lavender , W. H. Kaye , and C. E. Wierenga . 2019. “Task‐Switching Inefficiencies in Currently Ill, but Not Remitted Anorexia Nervosa.” International Journal of Eating Disorders 52, no. 11: 1316–1321. 10.1002/eat.23175. 31584714 PMC8127723

[eat24599-bib-0006] Black, C. M. D. , and G. Terence Wilson . 1996. “Assessment of Eating Disorders: Interview Versus Questionnaire.” International Journal of Eating Disorders 20, no. 1: 43–50. 10.1002/(SICI)1098-108X(199607)20:1<43::AID-EAT5>3.0.CO;2-4. 8807351

[eat24599-bib-0007] Braver, T. S. 2012. “The Variable Nature of Cognitive Control: A Dual Mechanisms Framework.” Trends in Cognitive Sciences 16, no. 2: 106–113. 10.1016/j.tics.2011.12.010. 22245618 PMC3289517

[eat24599-bib-0008] Breithaupt, L. , M. Slattery , M. Lauze , et al. 2025. “Study Protocol for a Randomized, Placebo‐Controlled, Double‐Masked Mechanistic Clinical Trial of Transdermal Estrogen Replacement in Hypoestrogenic Eating Disorders to Explore the Role of Estrogen on Cognitive Flexibility and Reward Processing.” Contemporary Clinical Trials 154: 107924. 10.1016/j.cct.2025.107924. 40294815 PMC12403050

[eat24599-bib-0009] Byrne, S. M. , K. L. Allen , A. M. Lampard , E. R. Dove , and A. Fursland . 2010. “The Factor Structure of the Eating Disorder Examination in Clinical and Community Samples.” International Journal of Eating Disorders 43, no. 3: 260–265. 10.1002/eat.20681. 19350647

[eat24599-bib-0010] Castro‐Fornieles, J. , E. de la Serna , A. Calvo , et al. 2019. “Functional MRI With a Set‐Shifting Task in Adolescent Anorexia Nervosa: A Cross‐Sectional and Follow‐Up Study.” Neuropsychologia 131, no. August: 1–8. 10.1016/j.neuropsychologia.2019.05.019. 31145908

[eat24599-bib-0011] Cooper, Z. , and C. Fairburn . 1987. “The Eating Disorder Examination: A Semi‐Structured Interview for the Assessment of the Specific Psychopathology of Eating Disorders.” International Journal of Eating Disorders 6, no. 1: 1–8. 10.1002/1098-108X(198701)6:1<1::AID-EAT2260060102>3.0.CO;2-9.

[eat24599-bib-0012] Delis, D. C. , E. Kaplan , and J. H. Kramer . 2001. Delis‐Kaplan Executive Function System (D‐KEFS). Examiner's Manual. https://www.scienceopen.com/document?vid=7379cf1a‐6f0e‐48b9‐aa9a‐897f1bad2f57.

[eat24599-bib-0013] Eddy, K. T. , and L. Breithaupt . 2023. “Atypical Anorexia Nervosa Diagnosis Should Exclude Those With Lifetime Anorexia Nervosa: Commentary on Walsh, Hagan, and Lockwood (2022).” International Journal of Eating Disorders 56, no. 4: 838–840. 10.1002/eat.23924. 36855014

[eat24599-bib-0014] Esteban, O. , C. J. Markiewicz , R. W. Blair , et al. 2019. “fMRIPrep: A Robust Preprocessing Pipeline for Functional MRI.” Nature Methods 16, no. 1: 111–116. 10.1038/s41592-018-0235-4. 30532080 PMC6319393

[eat24599-bib-0015] Forbush, K. T. , J. E. Wildes , L. O. Pollack , et al. 2013. “Development and Validation of the Eating Pathology Symptoms Inventory (EPSI).” Psychological Assessment 25, no. 3: 859–878. 10.1037/a0032639. 23815116

[eat24599-bib-0016] Garner, D. M. 2004. Eating Disorder Inventory‐3: Professional Manual. Vol. 35. Psychological Assessment Resources.

[eat24599-bib-0017] Girard, R. , E. Météreau , J. Thomas , M. Pugeat , C. Qu , and J.‐C. Dreher . 2017. “Hormone Therapy at Early Post‐Menopause Increases Cognitive Control‐Related Prefrontal Activity.” Scientific Reports 7, no. 1: 44917. 10.1038/srep44917. 28322310 PMC5359606

[eat24599-bib-0018] Gura‐Solomon, M. , R. Brener Yacobi , T. Kushnir , and E. Heled . 2024. “Cognitive Flexibility in Women Who Recovered From Anorexia Nervosa – A Model‐Based Approach.” Journal of Psychiatric Research 171, no. March: 38–42. 10.1016/j.jpsychires.2024.01.011. 38241968

[eat24599-bib-0019] Johnson‐Munguia, S. , S. Negi , Y. Chen , M. L. Thomeczek , and K. T. Forbush . 2024. “Eating Disorder Psychopathology, Psychiatric Impairment, and Symptom Frequency of Atypical Anorexia Nervosa Versus Anorexia Nervosa: A Systematic Review and Meta‐Analysis.” International Journal of Eating Disorders 57, no. 4: 761–779. 10.1002/eat.23989. 37317625 PMC13719182

[eat24599-bib-0020] Keshaviah, A. , K. Edkins , E. R. Hastings , et al. 2014. “Re‐Examining Premature Mortality in Anorexia Nervosa: A Meta‐Analysis Redux.” Comprehensive Psychiatry 55, no. 8: 1773–1784. 10.1016/j.comppsych.2014.07.017. 25214371

[eat24599-bib-0021] Kiesel, A. , M. Steinhauser , M. Wendt , et al. 2010. “Control and Interference in Task Switching—A Review.” Psychological Bulletin 136, no. 5: 849–874. 10.1037/a0019842. 20804238

[eat24599-bib-0022] Kray, J. , and U. Lindenberger . 2000. “Adult Age Differences in Task Switching.” Psychology and Aging 15, no. 1: 126–147. 10.1037/0882-7974.15.1.126. 10755295

[eat24599-bib-0023] Lang, K. , D. Stahl , J. Espie , J. Treasure , and K. Tchanturia . 2014. “Set Shifting in Children and Adolescents With Anorexia Nervosa: An Exploratory Systematic Review and Meta‐Analysis.” International Journal of Eating Disorders 47, no. 4: 394–399. 10.1002/eat.22235. 24347025

[eat24599-bib-0024] Lao‐Kaim, N. P. , L. Fonville , V. P. Giampietro , S. C. R. Williams , A. Simmons , and K. Tchanturia . 2015. “Aberrant Function of Learning and Cognitive Control Networks Underlie Inefficient Cognitive Flexibility in Anorexia Nervosa: A Cross‐Sectional fMRI Study.” PLoS One 10, no. 5: e0124027. 10.1371/journal.pone.0124027. 25970523 PMC4430209

[eat24599-bib-0025] Meng, X.‐l. , Robert Rosenthal , and Donald B. Rubin . 1992. “Comparing Correlated Correlation Coefficients.” Psychological Bulletin 111, no. 1: 172–175. 10.1037/0033-2909.111.1.172.

[eat24599-bib-0026] Miles, S. , I. Gnatt , A. Phillipou , and M. Nedeljkovic . 2020. “Cognitive Flexibility in Acute Anorexia Nervosa and After Recovery: A Systematic Review.” Clinical Psychology Review 81, no. November: 101905. 10.1016/j.cpr.2020.101905. 32891022

[eat24599-bib-0027] Miyake, A. , N. P. Friedman , M. J. Emerson , A. H. Witzki , A. Howerter , and T. D. Wager . 2000. “The Unity and Diversity of Executive Functions and Their Contributions to Complex ‘Frontal Lobe’ Tasks: A Latent Variable Analysis.” Cognitive Psychology 41, no. 1: 49–100. 10.1006/cogp.1999.0734. 10945922

[eat24599-bib-0028] Monsell, S. 2003. “Task Switching.” Trends in Cognitive Sciences 7, no. 3: 134–140. 10.1016/S1364-6613(03)00028-7. 12639695

[eat24599-bib-0029] Monteleone, A. M. , F. Pellegrino , G. Croatto , et al. 2022. “Treatment of Eating Disorders: A Systematic Meta‐Review of Meta‐Analyses and Network Meta‐Analyses.” Neuroscience & Biobehavioral Reviews 142, no. November: 104857. 10.1016/j.neubiorev.2022.104857. 36084848 PMC9813802

[eat24599-bib-0030] Phillipou, A. , U. Schmidt , E. Neill , S. Miles , P. McGorry , and K. T. Eddy . 2025. “Anorexia Nervosa—Facts, Frustrations, and the Future.” JAMA Psychiatry 82: 844–847. 10.1001/jamapsychiatry.2025.0812. 40465304

[eat24599-bib-0031] Rogers, R. D. , and S. Monsell . 1995. “Costs of a Predictible Switch Between Simple Cognitive Tasks.” Journal of Experimental Psychology: General 124, no. 2: 207–231. 10.1037/0096-3445.124.2.207.

[eat24599-bib-0032] Sato, Y. , N. Saito , A. Utsumi , et al. 2013. “Neural Basis of Impaired Cognitive Flexibility in Patients With Anorexia Nervosa.” PLoS One 8, no. 5: e61108. 10.1371/journal.pone.0061108. 23675408 PMC3651087

[eat24599-bib-0033] Schmitter‐Edgecombe, M. , and C. Sanders . 2009. “Task Switching in Mild Cognitive Impairment: Switch and Nonswitch Costs.” Journal of the International Neuropsychological Society 15, no. 1: 103–111. 10.1017/S1355617708090140. 19128533

[eat24599-bib-0034] Solmi, M. , F. Monaco , M. Højlund , et al. 2024. “Outcomes in People With Eating Disorders: A Transdiagnostic and Disorder‐Specific Systematic Review, Meta‐Analysis and Multivariable Meta‐Regression Analysis.” World Psychiatry 23, no. 1: 124–138. 10.1002/wps.21182. 38214616 PMC10785991

[eat24599-bib-0035] Spielberger, C. D. , S. J. Sydeman , A. E. Owen , and B. J. Marsh . 1999. “Measuring Anxiety and Anger With the State‐Trait Anxiety Inventory (STAI) and the State‐Trait Anger Expression Inventory (STAXI).” In The Use of Psychological Testing for Treatment Planning and Outcomes Assessment, 2nd ed. Lawrence Erlbaum Associates Publishers.

[eat24599-bib-0036] Tchanturia, K. , L. Giombini , J. Leppanen , and E. Kinnaird . 2017. “Evidence for Cognitive Remediation Therapy in Young People With Anorexia Nervosa: Systematic Review and Meta‐Analysis of the Literature.” European Eating Disorders Review 25, no. 4: 227–236. 10.1002/erv.2522. 28573705

[eat24599-bib-0037] Tchanturia, K. , N. Lounes , and S. Holttum . 2014. “Cognitive Remediation in Anorexia Nervosa and Related Conditions: A Systematic Review.” European Eating Disorders Review 22, no. 6: 454–462. 10.1002/erv.2326. 25277720

[eat24599-bib-0038] Uddin, L. Q. 2021. “Cognitive and Behavioural Flexibility: Neural Mechanisms and Clinical Considerations.” Nature Reviews Neuroscience 22, no. 3: 167–179. 10.1038/s41583-021-00428-w. 33536614 PMC7856857

[eat24599-bib-0039] Walsh, B. T. , K. E. Hagan , and C. Lockwood . 2023. “A Systematic Review Comparing Atypical Anorexia Nervosa and Anorexia Nervosa.” International Journal of Eating Disorders 56, no. 4: 798–820. 10.1002/eat.23856. 36508318

[eat24599-bib-0040] Wang, S. B. , E. K. Gray , K. A. Coniglio , et al. 2021. “Cognitive Rigidity and Heightened Attention to Detail Occur Transdiagnostically in Adolescents With Eating Disorders.” Eating Disorders 29, no. 4: 408–420. 10.1080/10640266.2019.1656470. 31675280 PMC7192764

[eat24599-bib-0041] Wonderlich, J. A. , M. Bershad , and J. E. Steinglass . 2021. “Exploring Neural Mechanisms Related to Cognitive Control, Reward, and Affect in Eating Disorders: A Narrative Review of FMRI Studies.” Neuropsychiatric Disease and Treatment 17, no. June: 2053–2062. 10.2147/NDT.S282554. 34188475 PMC8232881

[eat24599-bib-0042] Wylie, G. R. , E. A. Clark , P. D. Butler , and D. C. Javitt . 2010. “Schizophrenia Patients Show Task Switching Deficits Consistent With N‐Methyl‐D‐Aspartate System Dysfunction but Not Global Executive Deficits: Implications for Pathophysiology of Executive Dysfunction in Schizophrenia.” Schizophrenia Bulletin 36, no. 3: 585–594. 10.1093/schbul/sbn119. 18835838 PMC2879687

[eat24599-bib-0043] Zastrow, A. , S. Kaiser , C. Stippich , et al. 2009. “Neural Correlates of Impaired Cognitive‐Behavioral Flexibility in Anorexia Nervosa.” American Journal of Psychiatry 166, no. 5: 608–616. 10.1176/appi.ajp.2008.08050775. 19223435

