# Supplementary material for: Shared Versus Unique Features of Neural Activation During Cognitive Flexibility Across Restrictive Eating Disorder Presentations
Source: Int J Eat Disord. 2025 Nov 11;59(3):466–77. doi: 10.1002/eat.24599 (PMC12979975; doi:10.1002/eat.24599)
Supplement: Supplementary file 1 — Data S1: eat24599‐sup‐0001‐Supinfo.docx. [file EAT-59-466-s001.docx]

**Shared versus unique features of neural activation during cognitive flexibility across restrictive eating disorder presentations**

Adrienne L. Romer, Lauren Breithaupt, Meghan Slattery, Felicia Petterway, Lauren Lindman, Jason Scott, Meghan Lauze, Mia Cravitz, Zara Poon, Siddarth Seenivasa, Sarah Naticchia, Kristin N. Javaras, David Alperovitz, Jennifer J. Thomas, Elizabeth A. Lawson, Diego A. Pizzagalli, Franziska Plessow*, Poornima Kumar*, Madhusmita Misra*, and Kamryn T. Eddy*

* shared senior authorship.

**Supplementary Materials**

**Measures**

*Eating Disorders Examination Questionnaire (EDE).* A 28-item self-report questionnaire assessing the range, frequency and severity of eating disorder symptoms (Fairburn & Beglin, 2008). The EDE Restraint subscale scores were used as a measure of dietary restraint, with higher scores reflecting greater restraint.

*Eating Pathology Symptoms Inventory (EPSI).* A 45-item self-report questionnaire assessing eating disorder pathology (Forbush et al. 2013). The EPSI Cognitive Restraint and Dietary Restriction subscale scores were used as measures of cognitive restraint and restriction, respectively.

*Eating Disorders Inventory (EDI-3).* A 91-item self-report questionnaire assessing symptoms and psychological features of eating disorders (Garner, 2004). The EDI-3 Drive For Thinness subscale scores were used, with higher scores reflecting greater drive for thinness.

*Beck Depression Inventory (BDI-II).* A 21-item self-report questionnaire measuring characteristic attitudes and symptoms of depression (Beck et al., 1996). BDI total scores were used as a measure of depression symptoms, with higher scores indicating greater depression.

*State-Trait Anxiety Inventory-Trait (STAI-T).* A 20-item self-report questionnaire assessing trait anxiety (Spielberger, 1989). STAI-T total scores were used to assess trait anxiety symptoms, with higher scores indicating greater anxiety.

**Task-Switching fMRI Paradigm: Additional Task Details**

Stimulus duration was 1500 ms followed by an inter-trial interval (fixation cross) jittered between 500 ms and 4500 ms. Participants completed 5 blocks of each task condition (vowel-consonant discrimination, upper-lower case discrimination, task-switch) with 24 trials per block (360 trials total). Blocks were pseudo-randomly ordered into two sequences, with half of participants completing sequence 1 and half completing sequence 2, counterbalanced across participants (see Supplemental Methods for details). Specifically, each block was pseudo-randomly ordered according to a Latin square design such that each condition appeared only once at different serial positions within a block and single-task (vowel-consonant discrimination, upper-lower case discrimination) and task-switch conditions alternated (as in Girard et al. (2017)). The order of blocks was counterbalanced across participants. Participants practiced the task prior to completing it in the fMRI scanner.

Although mixing costs are traditionally calculated as the difference between performance in repeat trials during task-switching and performance in the single-task condition (Kiesel et al. 2010; Monsell 2003; Rogers and Monsell 1995), we calculated them as the performance difference between the task-switch (repeat + switch trials) and single-task conditions to match our fMRI task design (Girard et al., 2017). Additional analyses with the traditional mixing costs calculation are reported in the Supplement Result section.

**MRI Data Processing**

Results included in this manuscript come from preprocessing performed using *fMRIPrep* 20.2.1 (Esteban, Markiewicz, et al. (2018); Esteban, Blair, et al. (2018); RRID:SCR_016216), which is based on *Nipype* 1.5.1 (Gorgolewski et al. (2011); Gorgolewski et al. (2018); RRID:SCR_002502).

*Anatomical Data Preprocessing.* The T1-weighted (T1w) image was corrected for intensity non-uniformity (INU) with N4BiasFieldCorrection (Tustison et al. 2010), distributed with ANTs 2.3.3 (Avants et al. 2008, RRID:SCR_004757), and used as T1w-reference throughout the workflow. The T1w-reference was then skull-stripped with a *Nipype* implementation of the antsBrainExtraction.sh workflow (from ANTs), using OASIS30ANTs as target template. Brain tissue segmentation of cerebrospinal fluid (CSF), white-matter (WM) and gray-matter (GM) was performed on the brain-extracted T1w using fast (FSL 5.0.9, RRID:SCR_002823, Zhang, Brady, and Smith 2001). Brain surfaces were reconstructed using recon-all (FreeSurfer 6.0.1, RRID:SCR_001847, Dale, Fischl, and Sereno 1999), and the brain mask estimated previously was refined with a custom variation of the method to reconcile ANTs-derived and FreeSurfer-derived segmentations of the cortical gray-matter of Mindboggle (RRID:SCR_002438, Klein et al. 2017). Volume-based spatial normalization to two standard spaces (MNI152NLin6Asym) was performed through nonlinear registration with antsRegistration (ANTs 2.3.3), using brain-extracted versions of both T1w reference and the T1w template.

*Functional Data Preprocessing.* The following preprocessing was performed on the functional MRI data. First, a reference volume and its skull-stripped version were generated using a custom methodology of *fMRIPrep*. A deformation field to correct for susceptibility distortions was estimated based on *fMRIPrep*’s *fieldmap-less* approach. The deformation field resulted from co-registering the BOLD reference to the same-subject T1w-reference with its intensity inverted (Wang et al. 2017; Huntenburg 2014). Registration was performed with antsRegistration (ANTs 2.3.3), and the process regularized by constraining deformation to be nonzero only along the phase-encoding direction, and modulated with an average fieldmap template (Treiber et al. 2016). Based on the estimated susceptibility distortion, a corrected EPI (echo-planar imaging) reference was calculated for a more accurate co-registration with the anatomical reference. The BOLD reference was then co-registered to the T1w reference using bbregister (FreeSurfer) which implements boundary-based registration (Greve and Fischl 2009). Co-registration was configured with six degrees of freedom. Head-motion parameters with respect to the BOLD reference (transformation matrices, and six corresponding rotation and translation parameters) were estimated before any spatiotemporal filtering using mcflirt (FSL 5.0.9, Jenkinson et al. 2002). BOLD runs were slice-time corrected using 3dTshift from AFNI 20160207 (Cox and Hyde 1997, RRID:SCR_005927). The BOLD time-series were resampled onto the following surfaces (FreeSurfer reconstruction nomenclature): *fsaverage*. The BOLD time-series (including slice-timing correction when applied) were resampled onto their original, native space by applying a single, composite transform to correct for head-motion and susceptibility distortions. The BOLD time-series were resampled into standard space, generating a *preprocessed BOLD run in MNI152NLin6Asym space*. First, a reference volume and its skull-stripped version were generated using a custom methodology of *fMRIPrep*. Automatic removal of motion artifacts using independent component analysis (ICA-AROMA, Pruim et al. 2015) was performed on the *preprocessed BOLD on MNI space* time-series after removal of non-steady state volumes and spatial smoothing with an isotropic, Gaussian kernel of 6mm FWHM (full-width half-maximum). Corresponding “non-aggresively” denoised runs were produced after such smoothing. Several confounding time-series were calculated based on the *preprocessed BOLD*: framewise displacement (FD), DVARS and three region-wise global signals. FD and DVARS were calculated for each functional run, both using their implementations in *Nipype* (following the definitions by Power et al. 2014). Frames that exceeded a threshold of 0.5 mm FD or 1.5 standardised DVARS were annotated as motion outliers. All resamplings can be performed with *a single interpolation step* by composing all the pertinent transformations (i.e., head-motion transform matrices, susceptibility distortion correction when available, and co-registrations to anatomical and output spaces). Gridded (volumetric) resamplings were performed using antsApplyTransforms (ANTs), configured with Lanczos interpolation to minimize the smoothing effects of other kernels (Lanczos 1964). Non-gridded (surface) resamplings were performed using mri_vol2surf (FreeSurfer).

**Results**

*Whole-brain analyses of diagnostic group differences during task-switching*

Whole-brain one-way ANOVA demonstrated significant task condition x diagnostic group interactions in the left superior medial frontal cortex, partially overlapping with the dlPFC ROI, and in the left cerebellum Crus I/II and right medial orbitofrontal cortex during task-switch vs. single task conditions (Table S4 and Figure S1). We extracted BOLD estimates from these three clusters to conduct post-hoc independent-samples t-tests comparing neural activation between each pair of diagnostic group for task-switch and single-task conditions. Similar to the dlPFC ROI results, post-hoc t-tests revealed that these interactions were driven by differential BOLD response during the single-task condition between the AN and hx-AN groups. Specifically, the hx-AN group showed greater BOLD response within the left superior medial frontal cortex [*t*(62)=3.310, *p*=0.002] and right medial orbitofrontal cortex [*t*(62)=3.361, *p*=0.001] during the single-task condition than the AN group. The AN group showed significantly lower left cerebellar Crus I/II BOLD response during the single-task condition compared to the hx-AN group [*t*(62)=3.361, *p*=0.001]. These results were significant after FDR correction for the 18 post-hoc t-tests (q<0.05).

*Analyses with the traditional mixing costs calculation*

As our primary analyses calculated mixing costs as the difference in dlPFC activation/ performance between the task-switching (switch + repeat trials) and single task condition to be consistent with the fMRI task design adapted from Girard et al. (2017), we conducted our analyses with the traditional calculation of mixing costs (difference between repeat trials and single task) (Kiesel et al. 2010; Monsell 2003; Rogers and Monsell 1995). Consistent with our primary analyses in Table 1, there were no significant behavioral differences in the error rate (*F*(2,83)=0.673, *p*=0.513) or RT (*F*(2,83)=0.040, *p*=0.961) mixed costs across groups. When comparing left dlPFC activation during the repeat trials vs. single task across the groups, the task condition x group interaction was similar to the interaction reported in the main text (task-switch vs. single task): (*F*(2,83)=2.63, *p*=0.078), indicating our primary findings are robust to differences in methods for calculating mixing costs. Associations between left dlPFC BOLD during repeat trials vs. single task with EDE Dietary Restraint (β=0.293, *p*=0.008) and EPSI Cognitive Restraint scales (β=0.238, *p*=0.034) also were significant.

*Follow-Up Analyses of Association between EDE Dietary Restraint and dlPFC activation*

To test whether the association between EDE Dietary Restraint and left dlPFC activation was robust to a different measure of restraint symptoms, we additionally examined the association between EPSI Cognitive Restraint and left dlPFC activation during the task-switching vs. single-task condition. We found that greater left dlPFC BOLD during task-switch vs. single-task also was associated with higher EPSI Cognitive Restraint scores (Std. B=0.328, 95% CI [0.118, 0.538], *p*=0.003), suggesting consistency of findings across different measures of restraint. To determine whether this relation was driven by dlPFC activation during the task-switch or single-task condition, we tested this relation separately by condition (Table S3). Higher EPSI Cognitive Restraint was associated with lower left dlPFC BOLD during the single-task (β=-0.208, 95% CI [-0.427, 0.010], *p*=0.061) rather than task-switch condition (β=0.120, 95% CI [-0.102, 0.343], *p*=0.286), albeit non-significant, which also were different from each other (Meng's test: z=-3.11, *p*=0.001).

**References**

Avants, Brian B., Charles L. Epstein, Murray Grossman, and James C. Gee. 2008. “Symmetric

Diffeomorphic Image Registration with Cross-Correlation: Evaluating Automated Labeling of Elderly and Neurodegenerative Brain.” *Medical Image Analysis* 12 (1): 26-41.

https://doi.org/10.1016/j.media.2007.06.004

Beck, Aaron T., Robert A. Steer, and Gregory Brown. 1996. “Beck Depression Inventory–II (BDI-

II)”. APA PsycTests. https://doi.org/10.1037/t00742-000

Cox, Robert W., and James S. Hyde. 1997. “Software Tools for Analysis and Visualization of

fMRI Data.” *NMR in Biomedicine* 10 (4-5): 171–78. https://doi.org/10.1002/(SICI)1099-1492(199706/08)10:4/5<171::AID-NBM453>3.0.CO;2-L

Dale, Anders M., Bruce Fischl, and Martin I. Sereno. 1999. “Cortical Surface-Based Analysis: I.

Segmentation and Surface Reconstruction.” *NeuroImage* 9 (2): 179-94.

https://doi.org/10.1006/nimg.1998.0395

Esteban, Oscar, Christopher J. Markiewicz, Ross W. Blair, et al. 2019. “fMRIPrep: a robust

preprocessing pipeline for functional MRI." *Nature methods 16* (1): 111-116. https://doi.org/10.1038/s41592-018-0235-4

Fairburn Christopher G., and Sarah J. Beglin. 2008. “Eating disorder examination questionnaire.”

*Cognitive Behavior Therapy and Eating Disorders* 309 (313): 509–528.

Forbush, Kelsie T., Jennifer E. Wildes, Lauren O. Pollack, et al. 2013. “Development and Validation of the Eating Pathology Symptoms Inventory (EPSI).” *Psychological Assessment* 25 (3): 859–78. https://doi.org/10.1037/a0032639.

Garner, David M. 2004. "Eating disorder inventory-3 (EDI-3)." *Professional manual. Odessa, FL:*

*Psychological Assessment Resources* 1.

Girard, Romuald, Elise Météreau, Julie Thomas, Michel Pugeat, Chen Qu, and Jean-Claude Dreher. 2017. "Hormone therapy at early post-menopause increases cognitive control-related prefrontal activity." *Scientific reports* 7 (1): 44917. https://doi.org/10.1038/srep44917

Gorgolewski, Krzysztof, Christopher D. Burns, Cindee Madison, et al. 2011. "Nipype: a flexible,

lightweight and extensible neuroimaging data processing framework in python." *Frontiers in neuroinformatics* 5: 13. https://doi.org/10.3389/fninf.2011.00013

Gorgolewski, Krzysztof J., Oscar Esteban, Christopher J. Markiewicz, et al. 2018. "Nipype."

*Software*. https://doi.org/10.5281/zenodo.596855

Greve, Douglas N, and Bruce Fischl. 2009. “Accurate and Robust Brain Image Alignment Using

Boundary-Based Registration.” *NeuroImage* 48 (1): 63-72.

https://doi.org/10.1016/j.neuroimage.2009.06.060

Huntenburg, Julia M. 2014. “Evaluating Nonlinear Coregistration of BOLD EPI and T1w Images.”

Master’s Thesis, Berlin: Freie Universität. http://hdl.handle.net/11858/00-001M-0000-002B-1CB5-A

Jenkinson, Mark, Peter Bannister, Michael Brady, and Stephen Smith. 2002. “Improved

Optimization for the Robust and Accurate Linear Registration and Motion Correction of Brain Images.” *NeuroImage* 17 (2): 825–41. https://doi.org/10.1006/nimg.2002.1132

Kiesel, Andrea, Marco Steinhauser, Mike Wendt, et al. 2010. “Control and Interference in Task Switching—A Review.” *Psychological Bulletin* (US) 136 (5): 849–74. https://doi.org/10.1037/a0019842.

Klein, Arno, Satrajit S. Ghosh, Forrest S. Bao, et al. 2017. “Mindboggling Morphometry of

Human Brains.” *PLOS Computational Biology* 13 (2): e1005350. https://doi.org/10.1371/journal.pcbi.1005350

Lanczos, Cornelius. (1964). “Evaluation of Noisy Data.” *Journal of the Society for Industrial and*

*Applied Mathematics Series B Numerical Analysis* 1 (1): 76-85. https://doi.org/10.1137/0701007

Monsell, Stephen. 2003. “Task Switching.” *Trends in Cognitive Sciences* 7 (3): 134–40. https://doi.org/10.1016/S1364-6613(03)00028-7.

Power, Jonathan D., Anish Mitra, Timothy O. Laumann, Abraham Z. Snyder, Bradley L.

Schlaggar, and Steven E. Petersen. 2014. “Methods to Detect, Characterize, and Remove Motion Artifact in Resting State fMRI.” *NeuroImage* 84 (Supplement C): 320–41. https://doi.org/10.1016/j.neuroimage.2013.08.048

Pruim, Raimon H. R., Maarten Mennes, Daan van Rooij, Alberto Llera, Jan K. Buitelaar, and

Christian F. Beckmann. 2015. “ICA-AROMA: A Robust ICA-Based Strategy for Removing Motion Artifacts from fMRI Data.” *NeuroImage* 112 (Supplement C): 267-77.

https://doi.org/10.1016/j.neuroimage.2015.02.064

Rogers, Robert D., and Stephen Monsell. 1995. “Costs of a Predictible Switch between Simple Cognitive Tasks.” *Journal of Experimental Psychology: General* (US) 124 (2): 207–31. https://doi.org/10.1037/0096-3445.124.2.207.

Spielberger, Charles D. 1989. “State-Trait Anxiety Inventory: Bibliography (2nd ed.).” *Palo Alto,*

*CA: Consulting Psychologists Press.*

Treiber, Jeffrey Mark, Nathan S. White, Tyler Christian Steed, et al. 2016. “Characterization and

Correction of Geometric Distortions in 814 Diffusion Weighted Images.” *PLOS ONE* 11 (3):e0152472. https://doi.org/10.1371/journal.pone.0152472

Tustison, Nicholas J., Brian B. Avants, Philip. A. Cook, et al. 2010. “N4ITK: Improved N3 Bias

Correction.” *IEEE Transactions on Medical Imaging* 29 (6): 1310–20. https://doi.org/10.1109/TMI.2010.2046908

Wang, Sijia, Daniel J. Peterson, J. C. Gatenby, Wenbin Li, Thomas J. Grabowski, and Tara M.

Madhyastha. 2017. “Evaluation of Field Map and Nonlinear Registration Methods for Correction of Susceptibility Artifacts in Diffusion MRI.” *Frontiers in Neuroinformatics* 11. https://doi.org/10.3389/fninf.2017.00017

Zhang, Yongyue, Michael Brady, and Stephen Smith. 2001. “Segmentation of Brain MR Images

Through a Hidden Markov Random Field Model and the Expectation-Maximization Algorithm.” *IEEE Transactions on Medical Imaging* 20 (1): 45–57. https://doi.org/10.1109/42.906424

**Table S1.** *Bivariate correlations among all behavioral and symptom study variables.*

|  | 1 | 2 | 3 | 4 | 5 | 6 | 7 | 8 | 9 | 10 | 11 | 12 | 13 |
| --- | --- | --- | --- | --- | --- | --- | --- | --- | --- | --- | --- | --- | --- |
| 1. Age | 1 |  |  |  |  |  |  |  |  |  |  |  |  |
| 2. BMI | -.052 | 1 |  |  |  |  |  |  |  |  |  |  |  |
| 3. EDE Restraint | -.098 | -.114 | 1 |  |  |  |  |  |  |  |  |  |  |
| 4. EPSI Restraint | .006 | -.160 | .**495** | 1 |  |  |  |  |  |  |  |  |  |
| 5. EPSI Restrict | -.094 | **-.273** | **.274** | **.310** | 1 |  |  |  |  |  |  |  |  |
| 6. EDI Drive for Thinness | .087 | .131 | **.627** | **.531** | **.407** | 1 |  |  |  |  |  |  |  |
| 7. BDI-II | -.173 | -.039 | **.296** | -.012 | **.476** | **.499** | 1 |  |  |  |  |  |  |
| 8. STAI-T | -.103 | -.020 | **.279** | .035 | **.383** | **.500** | **.822** | 1 |  |  |  |  |  |
| 9. Mean FD | -.020 | **.311** | -.184 | -.104 | -.080 | .057 | .172 | .045 | 1 |  |  |  |  |
| 10. RT Mixed Costs (ms) | .209 | -.059 | .012 | .053 | **-.241** | .008 | -.150 | -.155 | .164 | 1 |  |  |  |
| 11. Error Rate Mixed Costs (%) | .012 | -.038 | **.314** | .167 | **.264** | .211 | .109 | .075 | -.195 | -.112 | 1 |  |  |
| 12. RT Switch Costs (ms) | .033 | **-.226** | .134 | .078 | .085 | .119 | .143 | .173 | -.082 | **.419** | .020 | 1 |  |
| 13. Error Switch Costs (%) | .006 | -.037 | .154 | .110 | .063 | .062 | -.018 | -.024 | -.013 | .046 | .109 | **.344** | 1 |

*Note.* Correlations shown in bold are significant at *p*<0.05. BMI=Body Mass Index; EDE=Eating Disorders Examination; EDI=Eating Disorders Inventory; EPSI=Eating Pathology Symptoms Inventory; BDI=Beck Depression Inventory; STAI-T=State-Trait Anxiety Inventory-Trait; RT=reaction time; TS=Task Switch.

**Table S2.** *Whole-Brain Main Effects of Task-Switching Across All Participants.*

| **Cluster Size (k)** | **Peak Region** | **MNI Coordinates** | | | **Peak T Score** |
| --- | --- | --- | --- | --- | --- |
|  |  | **x** | **y** | **z** |  |
| ***Task-Switch > Single Task*** | | | | | |
| 10,272 | Left Superior Parietal Lobule | -40 | -44 | 46 | 14.31 |
|  | Left Supramarginal Gyrus | -34 | -48 | 38 | 13.49 |
|  | Left Lateral Occipital Cortex | -26 | -68 | 48 | 13.01 |
| 13,479 | Left Precentral Gyrus | -46 | 2 | 36 | 13.57 |
|  | Left Middle Frontal Gyrus | -46 | 32 | 24 | 12.53 |
|  | Right Middle Frontal Gyrus | 28 | 2 | 54 | 12.42 |
| 3297 | Left Cerebellar Lobule VIIB | -28 | -72 | -50 | 11.20 |
|  | Right Cerebellar Lobule VI/Crus I | 10 | -76 | -24 | 10.07 |
|  | Left Cerebellar Crus II | -8 | -80 | -36 | 9.93 |
| 641 | Right Middle Temporal Gyrus | 56 | -54 | -8 | 9.87 |
|  | Right Inferior Temporal Gyrus | 52 | -50 | -24 | 5.75 |
| 73 | Right Thalamus | 8 | -14 | 10 | 8.40 |
| 234 | Right Cerebellar Lobule VI | 30 | -60 | -30 | 8.00 |
|  | Right Cerebellar Crus I | 36 | -68 | -28 | 6.66 |
| 545 | Left Intracalcarine Cortex | -12 | -76 | 10 | 7.13 |
|  | Right Intracalcarine Cortex | 12 | -68 | 10 | 7.03 |
|  | Left Cerebellar Lobule VI | -14 | -62 | -28 | 6.70 |
| 78 | Left Angular/Middle Temporal Gyrus | -52 | -52 | 12 | 6.91 |
| 57 | Right Caudate | 12 | 0 | 18 | 6.55 |
|  | Right Caudate | 10 | 6 | 8 | 6.32 |
| ***Single Task > Task-Switch*** | | | | | |
| 497 | Left Paracingulate Gyrus | -8 | 40 | -10 | 7.11 |
|  | Left Paracingulate Gyrus | -10 | 46 | -4 | 6.83 |
|  | Left Subcallosal Cortex | -6 | 26 | -12 | 6.74 |
| 50 | Left Posterior Cingulate | -4 | -46 | 28 | 7.02 |
| ***Switch > Repeat*** | | | | | |
| 4845 | Left Lateral Occipital Cortex | -22 | -68 | 54 | 8.31 |
|  | Left Lateral Occipital Cortex | -10 | -74 | 56 | 8.16 |
|  | Right Lateral Occipital Cortex | 12 | -64 | 58 | 7.44 |
| 2841 | Right Paracingulate Gyrus | 0 | 14 | 46 | 7.12 |
|  | Left Middle Frontal Gyrus | -30 | -2 | 66 | 7.08 |
|  | Left Supplementary Motor Cortex | -2 | 8 | 54 | 6.91 |
| 589 | Right Middle Frontal Gyrus | 30 | 0 | 60 | 6.44 |
|  | Right Superior Frontal Gyrus | 28 | -4 | 68 | 5.74 |
|  | Right Superior Frontal Gyrus | 24 | 4 | 68 | 4.64 |
| 2081 | Left Precentral Gyrus | -42 | 4 | 36 | 6.18 |
|  | Left Precentral Gyrus | -52 | 8 | 36 | 6.12 |
|  | Left Insular Cortex | -30 | 22 | 8 | 5.59 |
| 298 | Right Lateral Occipital Cortex | 42 | -86 | -4 | 5.18 |
|  | Right Occipital Pole | 30 | -96 | -4 | 5.04 |
|  | Right Occipital Pole | 16 | -100 | 2 | 4.31 |
| 354 | Left Lateral Occipital Cortex | -36 | -90 | -10 | 5.16 |
|  | Left Occipital Pole | -26 | -98 | -2 | 4.34 |
|  | Left Occipital Fusiform Gyrus | -22 | -90 | -18 | 4.23 |
| 163 | Right Insular Cortex | 32 | 22 | 6 | 4.82 |
|  | Right Frontal Operculum Cortex | 44 | 18 | -4 | 4.01 |
| 323 | Right Superior Parietal Lobule | 34 | -48 | 46 | 4.79 |
|  | Right Supramarginal Gyrus | 38 | -36 | 38 | 4.42 |
|  | Right Superior Parietal Lobule | 40 | -46 | 54 | 4.24 |
| 74 | Right Precentral Gyrus | 44 | 8 | 28 | 4.69 |
| 84 | Right Cerebellar Lobule VI | 34 | -40 | -36 | 4.67 |
|  | Right Cerebellar Lobule X | 30 | -38 | -44 | 4.45 |
|  | Right Cerebellar Lobule VI | 36 | -48 | -32 | 4.41 |
| 301 | Left Lateral Occipital Cortex | -48 | -70 | -10 | 4.59 |
|  | Left Lateral Occipital Cortex | -54 | -64 | -4 | 4.35 |
|  | Left Inferior Temporal Gyrus | -42 | -60 | -8 | 4.25 |
| ***Repeat > Switch*** | | | | | |
| 642 | Left Middle Temporal Gyrus | -66 | -18 | -6 | 6.14 |
|  | Left Middle Temporal Gyrus | -66 | -14 | -14 | 5.26 |
|  | Left Middle Temporal Gyrus | -62 | -24 | -18 | 4.73 |
| 532 | Right Middle Temporal Gyrus | 64 | -12 | -10 | 5.82 |
|  | Right Superior Temporal Gyrus | 68 | -16 | -2 | 5.41 |
|  | Right Middle Temporal Gyrus | 56 | -30 | -6 | 5.33 |
| 2118 | Left Paracingulate Gyrus | -4 | 50 | 4 | 5.62 |
|  | Left Frontal Pole | -10 | 58 | 30 | 5.51 |
|  | Left Superior Frontal Gyrus | -6 | 48 | 34 | 5.27 |
| 69 | Right Amygdala | 22 | -6 | -18 | 5.21 |
|  | Right Amygdala | 26 | -12 | -14 | 4.13 |
|  | Right Hippocampus | 32 | -14 | -22 | 3.36 |
| 152 | Left Frontal Orbital Cortex | -44 | 24 | -14 | 4.80 |
|  | Left Frontal Orbital Cortex | -30 | 16 | -20 | 4.26 |
|  | Left Frontal Orbital Cortex | -36 | 26 | -20 | 3.69 |
| 85 | Left Planum Polare | -44 | -14 | -4 | 4.66 |
| 78 | Right Temporal Pole | 46 | 14 | -18 | 4.62 |
|  | Right Temporal Pole | 48 | 18 | -30 | 4.34 |
|  | Right Temporal Pole | 42 | 10 | -34 | 4.08 |
| 70 | Left Cingulate Gyrus | -8 | -50 | 34 | 4.28 |
|  | Left Cingulate Gyrus | -6 | -50 | 20 | 3.72 |

*Note.* Clusters demonstrating significant main effects of task-switch vs. single-task conditions and switch vs. repeat trials within the task-switch condition are shown from a whole-brain t-test conducted within SPM 12. A significance threshold of *p*<0.05 family-wise error (FWE) cluster correction was employed for the task-switch vs. single-task contrast. An initial voxel forming threshold of uncorrected *p*<0.001 for the switch vs. repeat trials was employed with a significance threshold of p<0.05 FWE cluster correction.

**Table S3.** *Associations between Left dlPFC Activation during Task-Switch and Single Task Conditions and Symptom and Task Performance across all Participants.*

|  | **LDLPFC during TS** | | | **LDLPFC during Single Task** | | |
| --- | --- | --- | --- | --- | --- | --- |
|  | **Std. B** | **95% CI** | **P-Value** | **Std. B** | **95% CI** | **P-Value** |
| EDE Dietary Restraint | 0.191 | [-0.022, 0.403] | 0.078 | -0.086 | [-0.302, 0.130] | 0.430 |
| EPSI Cognitive Restraint | 0.120 | [-0.102, 0.343] | 0.286 | -0.208 | [-0.427, 0.010] | 0.061 |
| RT Mixed Costs (ms) | 0.144 | [-0.066, 0.354] | 0.177 | -0.005 | [-0.218, 0.207] | 0.961 |
| Error Rate Mixed Costs (%) | 0.054 | [-0.163, 0.271] | 0.622 | -0.151 | [-0.366, 0.064] | 0.167 |
| RT TS (ms) | 0.142 | [-0.073, 0.357] | 0.192 | -0.028 | [-0.245, 0.190] | 0.800 |
| Error Rate TS (%) | 0.064 | [-0.149, 0.277] | 0.551 | -0.100 | [-0.313, 0.112] | 0.350 |
| RT Single Task (ms) | 0.045 | [-0.164, 0.253] | 0.672 | -0.039 | [-0.248, 0.170] | 0.713 |
| Error Rate Single Task (%) | 0.024 | [-0.180, 0.227] | 0.817 | 0.072 | [-0.131, 0.276] | 0.482 |

*Note.* Standardized estimates and 95% confidence intervals (CIs) are shown from separate regression models of left dlPFC activation during Task-Switch and during Single-Task conditions relating to each of the variables in the first column. Age was included as a covariate. P-values are unadjusted. BMI=Body Mass Index; EDE=Eating Disorders Examination; EDI=Eating Disorders Inventory; EPSI=Eating Pathology Symptoms Inventory; BDI=Beck Depression Inventory; STAI-T=State-Trait Anxiety Inventory-Trait; RT=reaction time; TS=Task Switch.

**Table S4.** *Whole-Brain Differences in Neural Activation during Task-Switching vs. Single Task across Women with Current AN, History of AN, and Atypical AN.*

| **Cluster Size (k)** | **FWE-Cluser-Corrected P-Value** | **Peak Region** | **MNI Coordinates** | | | **Peak F Score** |
| --- | --- | --- | --- | --- | --- | --- |
|  |  |  | **x** | **y** | **z** |  |
| 229 | 0.001 | Left Superior Medial Frontal | -10 | 36 | 44 | 11.37 |
|  |  | Left Superior Medial Frontal | -6 | 46 | 50 | 8.70 |
|  |  | Left Superior Medial Frontal | -18 | 26 | 54 | 8.41 |
| 146 | 0.024 | Left Cerebellum Crus II | -18 | -80 | -34 | 10.74 |
|  |  | Left Cerebellum Crus I | -30 | -76 | -34 | 8.93 |
|  |  | Left Cerebellum Crus I | -42 | -76 | -34 | 8.85 |
| 130 | 0.045 | Right Medial Orbitofrontal Cortex | 14 | 50 | -10 | 10.20 |
|  |  | Right Superior Frontal | 18 | 52 | 2 | 9.83 |
|  |  | Right Superior Frontal | 20 | 56 | 12 | 9.32 |

*Note.* Clusters demonstrating significant main effects of group are shown from a whole-brain one-way ANOVA conducted within SPM 12. A significance threshold of *p*<0.05 family-wise error (FWE) cluster correction with an initial voxel forming threshold of uncorrected *p*<0.005 was employed. Age was included as a covariate.

**Figure S1.** *Diagnostic Group Differences in Left dlPFC Activation During* *Task-Switching vs. Single Task with Individual Data Points Plotted.*

*
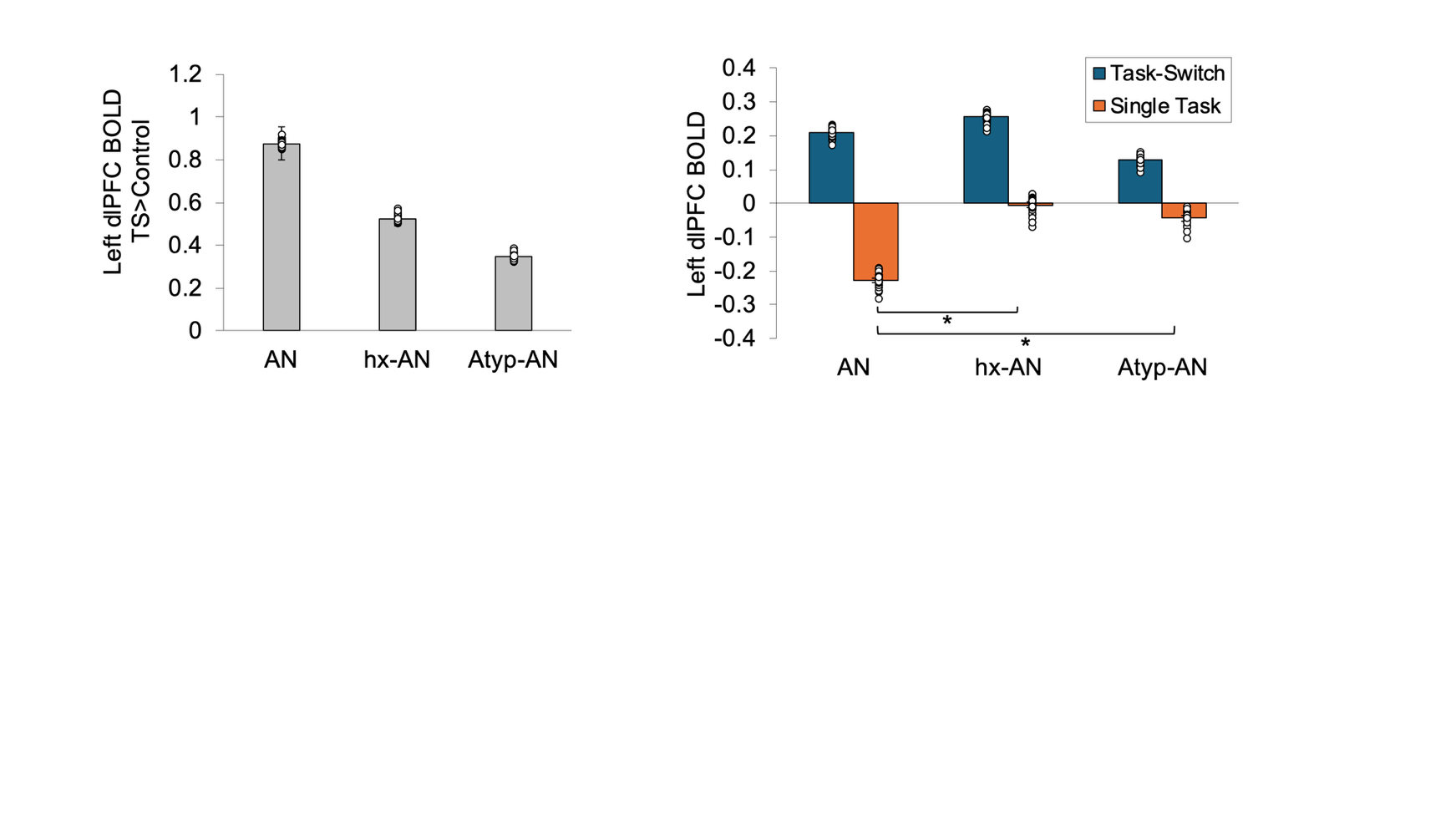
*

Bar charts are shown demonstrating significant diagnostic group differences in left dlPFC activation during task-switch vs. single task conditions (left panel) and the significant task condition x diagnostic group interaction (right panel). The AN group demonstrated a significantly greater difference in left dlPFC activation during task-switch vs. single task than the hx-AN and Atyp-AN groups. The task condition x diagnostic group interaction was driven by lower left dlPFC activation during the single-task condition in the AN group compared to the hx-AN and Atyp-AN groups. Between-group standard error bars +/- 2 are shown and individual data points are plotted over the bars.

**Figure S2.** *Whole-Brain Differences in Neural Activation During Task-Switching Across Diagnostic Groups.*


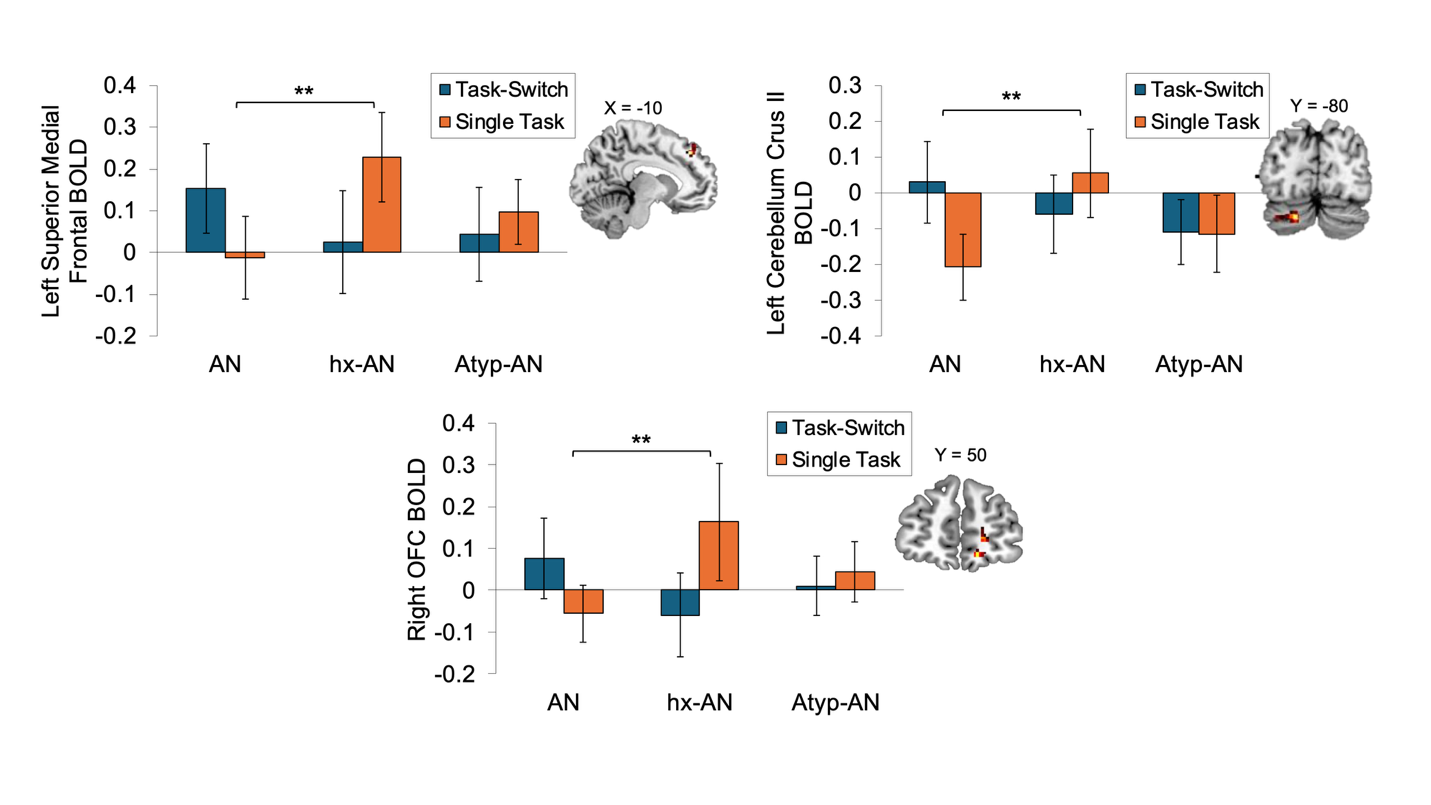
Bar charts depict significant main effects of group in the left superior medial frontal cortex (top left panel), left cerebellum Crus I/II (top right), and right medial orbitofrontal cortex (bottom) during task-switch vs. single-task conditions. Post-hoc independent-samples t-tests comparing neural activation between each pair of diagnostic groups are also shown for each bar chart. **unadjusted *p*<0.01.
